# Diagnostic routes and time intervals for ovarian cancer in nine international jurisdictions; findings from the International Cancer Benchmarking Partnership (ICBP)

**DOI:** 10.1038/s41416-022-01844-0

**Published:** 2022-05-26

**Authors:** Usha Menon, David Weller, Alina Zalounina Falborg, Henry Jensen, John Butler, Andriana Barisic, Anne Kari Knudsen, Rebecca J. Bergin, David H. Brewster, Victoria Cairnduff, Evangelia Ourania Fourkala, Anna T. Gavin, Eva Grunfeld, Elizabeth Harland, Jatinderpal Kalsi, Rebecca-Jane Law, Yulan Lin, Donna Turner, Richard D. Neal, Victoria White, Samantha Harrison, Irene Reguilon, Charlotte Lynch, Peter Vedsted, ICBP Module 4 Working Group, Andriana Barisic, Anna Gavin, Breann Hawryluk, Chantelle Anandan, Conan Donnelly, Henry Jensen, Jackie Boylan, Jacqueline Kelly, Kerry Moore, Maria Rejmyr Davis, Martin Malmberg, Mats Lambe, Oliver Bucher, Peter Vedsted, Rebecca Bergin, Sigrun Saur Almberg, Therese Kearney, Tindie Kalsi, Victoria Hammersley

**Affiliations:** 1 MRC Clinical Trials Unit at UCL, Institute of Clinical Trials & Methodology, University College London, London, UK; 2 Centre for Population Health Sciences, University of Edinburgh, Edinburgh, UK; 3 Research Unit for General Practice, Aarhus, Denmark; 4 The Royal Marsden NHS Foundation Trust, London, UK; 5 Ontario Health (Cancer Care Ontario), Toronto, Ontario Canada; 6 European Palliative Care Research Centre (PRC), Department of Oncology, Oslo, Norway; 7 University Hospital and Institute of Clinical Medicine, University of Oslo, Oslo, Norway; 8 Cancer Epidemiology Division, Cancer Council Victoria, Melbourne, VIC Australia; 9 Department of General Practice, University of Melbourne, Melbourne, VIC Australia; 10 Scottish Cancer Registry, Edinburgh, UK; 11 Northern Ireland Cancer Registry, Queen’s University Belfast, Belfast, UK; 12 Gynaecological Cancer Research Centre, Women’s Cancer, Institute for Women’s Health, University College London, London, UK; 13 Health Services Research Program, Ontario Institute for Cancer Research, Toronto, Ontario Canada; 14 Department of Epidemiology and Cancer Registry, CancerCare Manitoba, Winnipeg, Canada; 15 North Wales Centre for Primary Care Research, Bangor University, Wrexham, UK; 16 Population Oncology, Cancer Care Manitoba, Winnipeg, Canada; 17 Leeds Institute of Health Sciences, University of Leeds, Leeds, UK; 18 School of Psychology Deakin University, Geelong, VIC Australia; 19 Centre for Behavioral Research in Cancer, Cancer Council Victoria, Melbourne, VIC Australia; 20 International Cancer Benchmarking Partnership, Cancer Research UK, Stratford, UK; 21 Department of Patient Navigation, Cancer Care Manitoba, Winnipeg, Manitoba Canada; 22 Centre for Public Health, Queen’s University Belfast, Mulhouse Building, Belfast, UK; 23 Southern Sweden Regional Cancer Center, Medicon Village, Lund, Sweden; 24 Department of Oncology, Lund University Hospital, Lund, Sweden; 25 Regional Cancer Center Uppsala and Department of Medical Epidemiology and Biostatics, Karolinska Institutet, Stockholm, Sweden; 26 Department of Epidemiology and Cancer Registry, CancerCare Manitoba, Winnipeg, Manitoba Canada; 27 Centre for Behavioural Research in Cancer, Melbourne, VIC Australia; 28 Department of Cancer Research and Molecular Medicine, Faculty of Medicine, Norwegian University of Science and Technology, Trondheim, Norway; 29 Gynaecological Cancer Research Centre, Women’s Cancer, Institute for Women’s Health, University College London, London, UK

**Keywords:** Cancer, Health services

## Abstract

**Background:**

International Cancer Benchmarking Partnership Module 4 reports the first international comparison of ovarian cancer (OC) diagnosis routes and intervals (symptom onset to treatment start), which may inform previously reported variations in survival and stage.

**Methods:**

Data were collated from 1110 newly diagnosed OC patients aged >40 surveyed between 2013 and 2015 across five countries (51–272 per jurisdiction), their primary-care physicians (PCPs) and cancer treatment specialists, supplement by treatment records or clinical databases. Diagnosis routes and time interval differences using quantile regression with reference to Denmark (largest survey response) were calculated.

**Results:**

There were no significant jurisdictional differences in the proportion diagnosed with symptoms on the Goff Symptom Index (53%; *P* = 0.179) or National Institute for Health and Care Excellence NG12 guidelines (62%; *P* = 0.946). Though the main diagnosis route consistently involved primary-care presentation (63–86%; *P* = 0.068), onward urgent referral rates varied significantly (29–79%; *P* < 0.001). In most jurisdictions, diagnostic intervals were generally shorter and other intervals, in particular, treatment longer compared to Denmark.

**Conclusion:**

This study highlights key intervals in the diagnostic pathway where improvements could be made. It provides the opportunity to consider the systems and approaches across different jurisdictions that might allow for more timely ovarian cancer diagnosis and treatment.

## Background

Ovarian cancer (OC) is the eighth most common cancer in women globally and the gynaecological malignancy with the highest mortality, accounting for over 180,000 deaths per year [1]. At present, there is no effective screening for OC, and many women are diagnosed with late-stage disease, resulting in low survival rates [2, 3]. International variation persists in the proportion diagnosed at late stage, and in OC survival across all stages [2]. Exploring OC patient pathways in more depth may provide some insight into why this variation exists between countries, and why patients in some countries have more favourable outcomes.

OC often presents with symptoms which are non-specific and fairly common e.g. fatigue, bloating and non-specific abdominal pain [4]. The non-specific nature coupled with lower awareness of OC symptoms in the general public, makes diagnosis in primary-care challenging [5]. As 95% of women with OC report symptoms prior to diagnosis, earlier recognition of symptoms could improve timely diagnosis and hasten initiation of treatment which may impact on outcomes [6].

It needs to be noted that to date there is limited evidence on whether prolonged diagnostic and treatment intervals in OC are associated with poorer outcomes [7]. Initial reports suggest that once OC is symptomatic, a reduction in the time to diagnosis may not substantially impact survival or stage of the disease [8]. This is in keeping with data from the screening trial, UKCTOCS, where a significant increase in detection of early-stage disease with multimodal screening in asymptomatic women did not translate into a mortality benefit [9]. However, the evidence base is limited on this issue, and warrants further investigation that might help optimise the management of OC patients.

We undertook this study as part of the International Cancer Benchmarking Partnership Module 4 (ICBP M4), exploring variation in cancer outcomes across six countries (Australia, Canada, Denmark, Norway, Sweden and the UK). All have universal access to, and comparable expenditure on, healthcare and high-quality cancer registries. Across the ICBP countries, 5-year survival estimates for OC are typically below 45%, with lower survival reported in some countries such as the UK (37.1%), compared to Norway (46.2%), Australia (43.2%) and Canada (40.3%) [10]. Our aim was for OC patients to systematically compare the diagnostic routes and time intervals from first noticing symptoms to the start of treatment.

## Methods

**Fig. 1:**
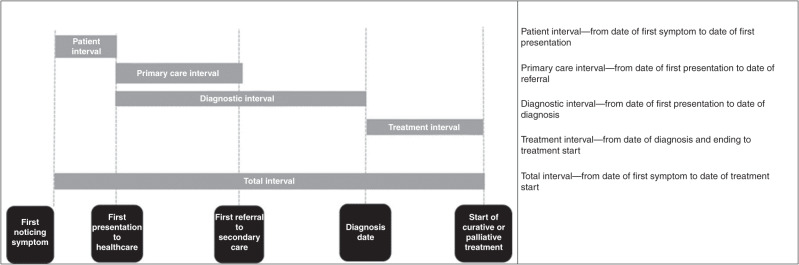
Time intervals measured as per the Aarhus Statement [13].

ICBP M4 methods have been previously reported [11]. Patients were identified through cancer registries in each of the nine jurisdictions: Victoria (Australia); Manitoba and Ontario (Canada); Denmark; Norway; Northern Ireland, England, Scotland and Wales (UK). Sweden was excluded as no OC data were available [12]. The target was to recruit 200 symptomatic patients recently diagnosed with ovarian cancer [11]. Routes to diagnosis for OCs were described using categories derived from the Aarhus Statement checklist, and time interval definitions were adapted from the Aarhus Statement (Fig. 1) [13].

All timepoints were validated manually if there was inconsistency (e.g. if the date of the first presentation occurred after treatment start) and negative time intervals were set to 0 days. Interval lengths were cut off at 365 days. Missing days were imputed based on specific rules to ensure that the direction of a possible misclassification bias was known (Supplementary File Appendix A).

### Identification of study population

Eligible patients were consecutive patients aged 40 years or more with a first diagnosis of OC including cancer in the fallopian tube and adnexa (ICD 10 codes: C56.9; C57.0–C57.9) [12]. Patients who previously had another non-index cancer were eligible, but those with synchronous cancers or previous history of OC were excluded. Patients diagnosed in the previous 3–6 months were eligible for contact by the jurisdictional cancer registry.

Patients underwent a vital status check, and were contacted through one of two routes:

1. A letter was sent to the relevant healthcare professional by the cancer registries, requesting that a pre-addressed envelope containing the questionnaire be forwarded to the patient, if they could confirm the patient was alive and aware of their diagnosis
2. A letter of invitation was sent directly by the registries or the research team to the patient.

### Data sources

Postal questionnaires were sent to identified patients, and with patient consent, their PCP and their Cancer Treatment Specialists (CTSs) (Supplementary File Appendix B). Survey data were supplemented with data from cancer registries and clinical databases. Data collected through questionnaires included routes to diagnosis, symptoms, treatment and socio-demographic characteristics and morbidity. Age, date of diagnosis and stage at diagnosis (tumour, node, metastasis (TNM) or Internationale Federation of Obstetrics and Gynaecology (FIGO) classification) were collected through cancer registries where available. The registry data were not available for Norway. The CTS data were not available for Northern Ireland and Manitoba.

### Data handling

Based on a standardised protocol, each jurisdiction established data collection procedures with the cancer registries, with adaptations to suit the local settings following initial pilot studies in some jurisdictions to assess survey acceptability and reliability [11]. Data cleaning was performed locally and centrally (Aarhus University) to ensure that the predefined set of rules was applied on the full dataset. Data queries were discussed with the local lead/team. Patients where age, date of diagnosis or date of consent were unknown were excluded.

The rules indicate which data source (patient, PCP, CTS, registry) should take precedence where responses between sources differed and included imputation rules based on the available data. The exact rule was guided by the measure in question—for example, patient interval was collected primarily from the patient questionnaire whereas primary-care timepoints were collected from the PCP questionnaire. All the measures were further validated using algorithms for outliers and out of range responses (e.g. negative time intervals). Predefined rules including a data ‘hierarchy’ regarding these information sources were used to calculate the route and time intervals and were based on the Aarhus Statement (Supplementary File Appendix A) [13].

### Covariates

The self-reported general health item from the 36-Item Short-Form Health Survey (SF36) was used to assess the health status of the patients [14]. Comorbidity was assessed as the presence of four major conditions (stroke, diabetes, lung or heart diseases) and categorised into: ‘none’, ‘medium’ (one or two), ‘high’ (three or four). Educational level was categorised as secondary or equivalent (lower) and university or equivalent (higher). Symptoms reported were divided into two categories: ‘ovarian-specific’ or ‘other’ symptoms (symptoms that were not among the six most frequently reported and undefined symptoms), in order to identify symptoms triggering clinical suspicion of OC. It was based on symptom coding done independently by two PCP-authors (PV and DW) using Goff Symptom Index (GSI) and NICE. Ovarian cancer: recognition and initial management NICE Guidelines. Clinical guideline 122 (NG122) [15, 16].

### Statistical analysis

Sample size considerations were based on the analysis of longer time intervals (more than the 75th centile) across ten jurisdictions. Wales was chosen as a reference point as this jurisdiction was expected to have most patients having the longest time interval (defined as the largest 75th centile). The proportion with the ‘short’ interval from the reference jurisdiction was compared with the proportions with ‘short’ intervals from the rest of the jurisdictions—that is, nine comparisons were performed. The sample size calculation was based on sample size determination for comparing proportions by χ^2^ test in contingency tables. We adjusted the method to accommodate our intention to undertake only nine comparisons. With a power of 90%, the method revealed a requirement for an overall sample of size 2000—that is, 200 patients in each of the 10 jurisdictions.

Quantile regression was used to estimate differences in intervals between all jurisdictions [17]. We compared the 50th (median), 75th and 90th percentiles. Denmark was chosen as the reference as it had the highest number of respondents, as well as one of the higher survival estimates relative to the other ICBP jurisdictions (following Australia and Norway) [10]. Counting days, we used the ‘qcount’ procedure [18]. The jittering process was applied for artificial smoothing of the data by adding a uniformly distributed noise to the count variable. Parameters were calculated with 1000 jittered. The differences in intervals between jurisdictions were calculated as marginal effects after quantile regression by setting the continuous covariate age to its mean value and the categorical covariates (gender and comorbidity) to their modes. The significance level was set to 0.05, and 95% confidence intervals (95%CI) were calculated when appropriate. Statistical analyses were carried out using STATA v14 software.

### Sensitivity and validity analyses

All analyses were undertaken using received questionnaires for eligible patients and additional sensitivity analyses were carried out on those completed within the 6-month and 9-month window from diagnosis to questionnaire, as per protocol. To estimate the effect of using patient-reported intervals only, a sensitivity analysis based solely on patient data was performed. The effect of excluding patients for whom at least one-time interval had not been reported was also investigated.

For dates of the first presentation to primary care, diagnosis and treatment, the agreement between the different data sources (patient, PCP, CTS and registry) was assessed by Lin’s concordance correlation coefficient (CCC) [19].

## Results

Between May 2013 and November 2015, 3204 OC patients were identified as eligible for the study across the nine participating jurisdictions. Of these, 84.8% (2716/3204) were contacted either directly or via their PCP (Supplementary Table 1). A total of 1221 patients (45.0% of contacted, 38.1% of eligible) completed the questionnaire. The response rate varied between jurisdictions, with the lowest in Norway at around 20% and the highest in Denmark at around 70% (Supplementary Table 1). Respondents were more likely to be younger, have less advanced disease and be alive at 1-year follow-up than non-responders (Supplementary Table 2).

Overall, 1110 patients were included in the analyses, equating to 34.6% (1110/3204) of all eligible patients. The reasons for the exclusion of 111 patients are detailed in Supplementary Table 1. Manitoba and Northern Ireland were only able to identify 143 patients each who were eligible to be included in the study, and only Denmark and England recruited more than the target 200 patients per jurisdiction. Of patients whose data were included in the analysis, 68% also had data from their PCP and 38% had data from their CTS.

### Baseline characteristics

The characteristics of the OC patients analysed for this study are shown in Table 1. The majority were in good health (82%) with no comorbidity (73%). The cohort was predominantly White (97%), with a median age of 64 years (interquartile range (IQR) 56, 71). More than half (55%) had never smoked and 72% of patients were categorised as having low levels of education. Data on histological subtypes were only available in the subgroup (38%; 426/1110) where the CTS had completed a questionnaire or information was provided by the registry (Supplementary Table 3). The majority (65%; 279/426) of the cases were invasive serous epithelial cancer, 3% (12/426) were endometrioid, clear cell and mucinous and 23% (100/426) were borderline ovarian neoplasms. About 40% of patients were diagnosed with early-stage disease (TNM and FIGO 2003 Stage I and II), ranging from around 30% in Northern Ireland to 45% in Victoria (low percentage in Norway but excluded due to low sample size). The proportion of missing stage data varied across jurisdictions, from 0% in Victoria to 32% in Ontario (84% in Norway but excluded due to the small sample size). Availability of treatment data was also variable but more complete for surgery and chemotherapy (85% of patients received surgery; 83% received chemotherapy).

**Table 1:** The characteristics of patients included in analyses aged 40 or over with the first diagnosis of ovarian cancer included in the analyses *n* (%).

	Denmark	England	Victoria	Scotland	Ontario	Wales	N Ireland	Manitoba	Norway	Total
No. of women	271	230	127	101	99	90	85	56	51	1110
Patient responses (% of eligible patients)	271 (69.8%)	256 (25.9%)	136 (44.9%)	140 (31.7%)	109 (24.9%)	98 (22.3%)	95 (66.4%)	56 (39.2%)	51 (14.2%)	1213 (37.8%)
Date first patient completed survey	05/11/2013	28/01/2013	11/07/2013	11/12/2013	30/06/2014	11/10/2013	08/08/2013	31/05/2013	04/10/2014	28/01/2013
Date last patient completed survey	06/11/2014	15/03/2015	19/03/2015	02/02/2015	22/06/2015	09/12/2014	22/12/2015	08/06/2015	18/10/2015	22/12/2015
Time interval from diagnosis to survey completion in months, median (IQI)	4 (3, 5)	4 (3, 5)	5 (4, 6)	6 (4, 8)	7 (6, 9)	5 (4, 7)	4 (3, 5)	6 (6, 7)	8 (7, 10)	5 (4, 6)
Survey completion within 6 months from diagnosis, *n* (%)	255 (94)	199 (87)	95 (75)	54 (53)	38 (38)	55 (61)	85 (100)	22 (39)	1 (2)	804 (72)
Age, years										
Median (IQI)	67 (58, 73)	64 (56, 72)	61 (53, 67)	62 (53, 69)	59 (52, 68)	67 (58, 72)	65 (55, 70)	60 (55, 67)	68 (55, 73)	64 (56, 71)
Health state										
Good	221 (82)	184 (80)	112 (88)	79 (78)	89 (90)	74 (82)	66 (78)	46 (82)	43 (84)	914 (82)
Fair	38 (14)	35 (15)	11 (9)	15 (15)	5 (5)	15 (17)	13 (15)	7 (13)	6 (12)	145 (13)
Poor	7 (3)	*n* ≤ 11	*n* ≤ 5	*n* ≤ 5	*n* ≤ 5	*n* ≤ 5	*n* ≤ 5	*n* ≤ 5	*n* ≤ 5	40 (4)
Missing	5 (2)	*n* ≤ 11	*n* ≤ 5	*n* ≤ 5	*n* ≤ 5	*n* ≤ 5	*n* ≤ 5	*n* ≤ 5	*n* ≤ 5	11 (1)
Comorbidity^a^										
No	185 (68)	168 (73)	99 (78)	79 (79)	78 (79)	60 (67)	67 (79)	36 (64)	41 (80)	813 (73)
Medium	86 (32)	60 (26)	26 (20)	20 (20)	18 (18)	29 (32)	18 (21)	18 (32)	9 (18)	284 (26)
High	0	*n* ≤ 5	*n* ≤ 5	*n* ≤ 5	*n* ≤ 5	*n* ≤ 5	0	*n* ≤ 5	*n* ≤ 5	6 (1)
Missing	0	*n* ≤ 5	*n* ≤ 5	*n* ≤ 5	*n* ≤ 5	*n* ≤ 5	0	*n* ≤ 5	*n* ≤ 5	7 (1)
Education										
Low	190 (70)	191 (83)	83 (65)	68 (67)	65 (66)	70 (78)	57 (67)	39 (70)	33 (65)	796 (72)
High	47 (17)	30 (13)	*n* ≤ 44	25 (25)	*n* ≤ 34	11 (12)	15 (18)	*n* ≤ 17	*n* ≤ 18	230 (21)
Missing	34 (13)	8 (4)	*n* ≤ 44	8 (8)	*n* ≤ 34	9 (10)	13 (15)	*n* ≤ 17	*n* ≤ 18	84 (8)
Ethnicity										
White	262 (97)	226 (98)	120 (94)	101 (100)	89 (90)	*n* ≤ 90	*n* ≤ 85	51 (91)	51 (100)	1072 (97)
Other	*n* ≤ 9	*n* ≤ 5	7 (6)	0	10 (10)	*n* ≤ 90	*n* ≤ 85	*n* ≤ 5	0	28 (4)
Missing	*n* ≤ 9	*n* ≤ 5	0	0	0	0	0	*n* ≤ 5	0	10 (1)
Smoking										
Currently	*n* ≤ 33	11 (5)	*n* ≤ 10	*n* ≤ 11	*n* ≤ 5	*n* ≤ 5	*n* ≤ 10	*n* ≤ 7	6 (12)	86 (8)
In the past	102 (38)	81 (35)	43 (34)	35 (35)	42 (42)	31 (34)	27 (32)	22 (39)	20 (39)	403 (36)
Never	136 (50)	138 (60)	74 (58)	55 (54)	54 (55)	55 (61)	48 (56)	27 (48)	25 (49)	612 (55)
Missing	*n* ≤ 33	0	*n* ≤ 10	*n* ≤ 11	*n* ≤ 5	*n* ≤ 5	*n* ≤ 10	*n* ≤ 7	0	9 (1)
Tumour stage—TNM & FIGO										
I	73 (27)	63 (27)	31 (25)	30 (30)	19 (19)	26 (29)	22 (26)	*n* ≤ 15	*n* ≤ 5	279 (25)
II	10 (4)	25 (11)	26 (20)	15 (15)	8 (8)	6 (7)	*n* ≤ 6	6 (11)	*n* ≤ 5	100 (9)
III	101 (37)	103 (45)	64 (50)	35 (35)	32 (32)	32 (36)	51 (60)	*n* ≤ 30	*n* ≤ 5	449 (40)
IV	45 (17)	31 (13)	6 (5)	15 (15)	8 (8)	10 (11)	6 (7)	*n* ≤ 5	*n* ≤ 5	129 (12)
Missing	42 (16)	8 (3)	0	6 (6)	32 (32)	16 (18)	*n* ≤ 6	*n* ≤ 5	43 (84)	153 (14)
Treatment surgery										
Yes	233 (86)	172 (75)	121 (95)	81 (80)	97 (98)	76 (84)	65 (76)	53 (95)	44 (86)	942 (85)
No	15 (6)	22 (10)	*n* ≤ 5	9 (9)	*n* ≤ 5	*n* ≤ 5	11 (13)	*n* ≤ 5	*n* ≤ 5	64 (6)
Missing	23 (8)	36 (16)	*n* ≤ 5	11 (11)	*n* ≤ 5	*n* ≤ 15	9 (11)	*n* ≤ 5	*n* ≤ 5	104 (9)
Treatment chemo										
Yes	220 (81)	190 (83)	115 (91)	83 (82)	87 (88)	66 (73)	72 (85)	53 (95)	39 (76)	925 (83)
No	19 (7)	14 (6)	*n* ≤ 15	8 (8)	12 (12)	*n* ≤ 5	*n* ≤ 10	*n* ≤ 5	*n* ≤ 5	83 (7)
Missing	32 (12)	26 (11)	*n* ≤ 5	10 (10)	0	*n* ≤ 20	*n* ≤ 5	*n* ≤ 5	*n* ≤ 10	102 (9)
Treatment radio										
Yes	0	0	*n* ≤ 5	0	*n* ≤ 5	*n* ≤ 5	*n* ≤ 5	*n* ≤ 5	*n* ≤ 5	20 (2)
No	120 (44)	100 (43)	98 (77)	44 (43)	89 (90)	*n* ≤ 40	51 (60)	38 (68)	*n* ≤ 25	595 (54)
Missing	151 (56)	130 (57)	*n* ≤ 25	57 (56)	*n* ≤ 5	51 (57)	*n* ≤ 35	*n* ≤ 20	29 (57)	495 (45)
Treatment other										
Yes^b^	0	3 (1)	0	*n* ≤ 5	*n* ≤ 5	1	*n* ≤ 5	*n* ≤ 5	*n* ≤ 10	13 (1)
No	271 (100)	78 (34)	82 (65)	*n* ≤ 40	95 (96)	33 (37)	43 (51)	*n* ≤ 5	*n* ≤ 5	648 (58)
Missing	0	149 (65)	45 (35)	60 (59)	*n* ≤ 5	56 (62)	*n* ≤ 45	54 (96)	40 (78)	449 (40)

*IQI* interquartile interval, *n/a* not applicable.

^a^Comorbidity coded as none = no reported, medium = 1–2 reported and high = 3+ reported.

^b^Includes antiVEGFdrugs, hormones and clinical trial.

Some numbers are not shown due to the data protection regulations.

### Routes to diagnosis

Table 2 illustrates the routes to diagnosis. Across the jurisdictions the predominant route to diagnosis was initiated by a visit to the PCP (70% of patients), with 68% obtaining an appointment within 0–6 days, 15% within 1–4 weeks and only 2% requiring to wait >4 weeks for a PCP appointment (Supplementary Table 4). Overall, 10% were diagnosed whilst being investigated for another disorder. A higher proportion of the patients in Canada (Manitoba and Ontario) and Northern Ireland were diagnosed via the A&E route (either presenting directly or following a visit to their PCP) compared to other jurisdictions (36% in Manitoba, around 25% in Northern Ireland and 22% in Ontario, compared to 8–15% elsewhere). Based on PCP data, the proportion of patients referred urgently varied significantly (29–79%; *P* < 0.001).

**Table 2:** Routes to diagnosis of ovarian cancer patients included in the analyses for each jurisdiction *n* (%).

	Denmark	England	Victoria	Scotland	Ontario	Wales	N Ireland	Manitoba	Norway	Total
No. of women	271	230	127	101	99	90	85	56	51	1110
Visit PCP	202 (75)	166 (72)	94 (74)	77 (76)	60 (61)	64 (71)	58 (68)	30 (54)	29 (57)	780 (70)
Visit PCP and Emergency department (A&E)^a^	6 (2)	13 (6)	8 (6)	*n* ≤ 5	10 (10)	7 (8)	15 (18)	14 (25)	*n* ≤ 5	82 (7)
Emergency department (A&E)^a^	15 (6)	*n* ≤ 10	9 (7)	*n* ≤ 5	12 (12)	*n* ≤ 5	5 (6)	6 (11)	*n* ≤ 5	63 (6)
Investigation for another problem	28 (10)	26 (11)	13 (10)	11 (11)	10 (10)	9 (10)	*n* ≤ 5	6 (11)	9 (18)	114 (10)
Symptomatic patients with missing route of diagnosis^b^	12 (4)	12 (5)	*n* ≤ 5	*n* ≤ 5	*n* ≤ 5	*n* ≤ 5	*n* ≤ 5	0	7 (14)	49 (4)
Other	8 (3)	*n* ≤ 5	*n* ≤ 5	0	*n* ≤ 5	*n* ≤ 5	*n* ≤ 5	0	*n* ≤ 5	22 (2)

^a^A&E: Accident and Emergency Department or Casualty.

^b^Routes to diagnosis were not reported in either of the data sources, but patient reported at least one symptom or date of first symptom.

Some numbers are not shown due to the data protection regulations.

### Symptoms prompting visit to physician

A median number of 2 (IQR 1–4) symptoms were reported by patients across jurisdictions (Supplementary Table 5). The most frequent patient-reported symptoms were ‘swelling in the abdomen, increased abdomen size, bloating or unexplained weight gain’ (52%), followed by ‘unexplained pain in the abdomen, stomach or pelvis’ (41%), or ‘fatigue’ (29%). Half the cohort also reported other symptoms, either not among the six most frequently reported, or undefined symptoms. Overall, 8% of patients reported that they experienced no symptoms prior to diagnosis, although there was some variation across jurisdictions. The patient-reported symptom profile was identical when we limited the analysis to only the 537 patients whose PCP had also completed the questionnaire (Supplementary Table 5b).

PCPs reported a similar but not identical symptom profile, and the data were derived from a sample size of approximately half that of the patient cohort. Across jurisdictions, PCPs reported a median of 1 (IQR 1–2) symptom at first presentation, with ‘unexplained pain in the abdomen, stomach or pelvis’ being the most common (35%), followed by ‘swelling in the abdomen, increased abdomen size, bloating or unexplained weight gain’ (22%) and ‘change in bowel habits’ (12%). A substantial proportion of cases were classed as having ‘other’ symptoms (39%). As expected, only a small number were categorised by the PCP as having no symptoms at presentation (overall 2% for all jurisdictions, although 15% in Manitoba).

The time intervals observed across jurisdictions are summarised in Table 3. The median total interval ranged from 57 days in Victoria to 125 days in Northern Ireland. Three jurisdictions had median total intervals of between 57 and 66 days, while four jurisdictions had intervals of between 110 and 125 days in length. At the 90th percentile, the total interval ranged from 246 days in Denmark to 365 days in Northern Ireland; four jurisdictions had intervals of between 311 and 339 days in length.

**Table 3:** A descriptive table with the different time intervals (days) for each of the nine jurisdictions depicted as 50th (median), 75th and 90th percentiles^a^.

	Denmark	England	Victoria	Scotland	Ontario	Wales	N Ireland	Manitoba	Norway
Patient interval									
Number	246	223	117	95	91	82	81	48	39
Median	12	27	28	21	33	31	35	23	11
75th percentile	47	60	83	62	82	61	75	101	41
90th percentile	125	171	232	254	151	194	187	365	61
Primary-care interval									
Number	164	161	66	64	29	55	58	32	7
Median	1	7	6	13	13	8	7	19	13
75th percentile	12	24	21	32	49	37	24	56	31
90th percentile	62	50	91	52	258	131	72	209	49
Diagnostic interval									
Number	244	219	116	94	86	84	77	48	38
Median	56	51	25	29	48	55	68	55	32
75th percentile	115	83	47	56	96	93	126	132	86
90th percentile	195	151	133	123	166	179	198	232	245
Treatment interval									
Number	269	226	125	100	98	88	84	55	49
Median	0	8	0	38	4	2	0	1	19
75th percentile	1	29	6	59	28	33	26	27	36
90th percentile	25	51	20	89	53	69	40	35	60
Total interval									
Number	225	210	107	77	88	81	76	44	35
Median	66	104	57	118	110	120	125	90	65
75th percentile	133	165	138	183	173	219	229	174	127
90th percentile	246	311	261	339	282	328	365	328	280

^a^See Fig. 1 for definitions of time intervals.

The median patient interval in most jurisdictions was between 21 and 35 days, except in Norway (11 days) and Denmark (12 days). The median primary-care interval ranged from 1 day in Denmark to 19 days in Manitoba, with four jurisdictions having interval lengths of around one week and three around two weeks. The median diagnostic interval ranged from 25 days in Victoria to 68 days in Northern Ireland, with three jurisdictions having intervals between 25 and 32 days, and four between 51 and 55 days. The median treatment interval was between 0 and 8 days in all jurisdictions except for Norway (19 days) and Scotland (38 days).

### Comparison of intervals between jurisdictions

**Fig. 2:**
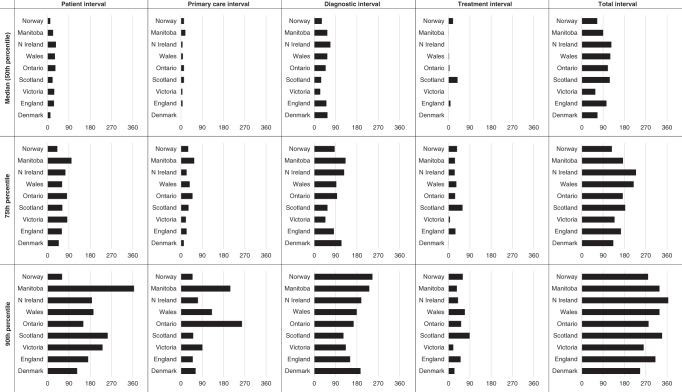
Graphs of differing interval lengths across jurisdictions for the 50th (median), 75th and 90th percentiles across all nine jurisdictions (days).

Table 4 and Fig. 2 show the differences in adjusted intervals across jurisdictions compared to the reference, Denmark. Except for the patient intervals in Norway, Ontario and Scotland, the patient and primary-care intervals in all other jurisdictions (across all percentiles) were significantly longer compared to Denmark. Treatment intervals were also significantly longer compared to Denmark across all jurisdictions and percentiles, except for Victoria. This resulted in total intervals that were significantly longer compared to Denmark across all jurisdictions, except for Victoria. Conversely, seven of the eight jurisdictions compared to Denmark show shorter median diagnostic intervals, with significantly shorter intervals across all percentiles in Victoria and Scotland.

**Table 4:**
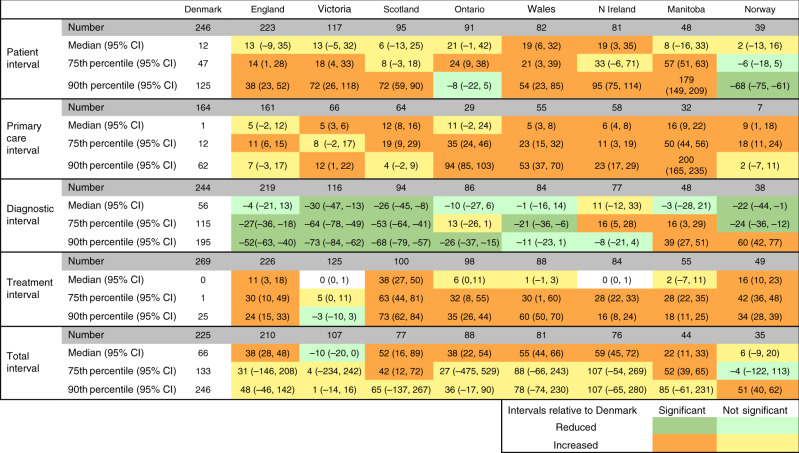
Difference in intervals for the 50th (median), 75th and 90th percentiles between Denmark (as the reference, the actual number of days included) and the other eight jurisdictions (days)^a^.

^a^See Fig. 1 for definitions of time interval.

For intervals, relative to Denmark. Orange: significantly increased; dark green: significantly reduced; light green: non-significantly reduced, yellow: non-significantly increased.

### Sensitivity and validity analyses

The estimates of routes to diagnosis, time intervals, and regression analysis trends were not significantly altered by changing the cut-off to 6 or 9 months, or using only patient data, or using only patients for whom all time intervals had been reported (results not shown). Comparing the dates between the different data sources showed adequate agreement between all data sources for all categories of dates (CCC = 0.90 for date of treatment, CCC ≥ 0.95 for date of diagnosis, CCC = 0.93 for date of first presentation to primary care).

## Discussion

To our knowledge, this is the first study to explore variation in routes to diagnosis and key time intervals in OC across multiple countries. Our study demonstrates that despite similar symptom profiles, there were important international differences across all intervals from symptom onset to treatment in OC. These variations were most pronounced for diagnostic and treatment intervals. Diagnostic intervals ranged from 25–68 days and were generally shorter than the reference jurisdiction, Denmark. Treatment intervals ranging from 0 to 38 days were longer for most jurisdictions compared to Denmark. The resulting variation in the total interval between jurisdictions was most obvious for the 75th and 90th percentiles. Ten percent of patients who waited longest had substantially longer total intervals (on average 209 days) compared to the median (95 days) for the whole cohort. There were also differences in use of urgent referrals that warrant further exploration. The variation in primary-care intervals suggest that improvements to primary-care referral processes, might help to improve the total interval.

Although some comparisons were limited by insufficient power due to low patient numbers, variation seen in time intervals broadly fall in line with observed international variation for OC survival. As demonstrated by the ICBP SurvMark-2 benchmark for patients diagnosed in 2010–2014, Denmark and Australia (42.1% and 43.2%) had higher 5-year OC survival whilst the UK had lower survival (37.1%) [10]. This follows the pattern seen in time intervals, with Denmark and Victoria (Australia) having the shortest median total intervals (66 days and 57 days respectively). Norway had the highest OC 5-year survival (46.2%) for same period and one of the shortest total median intervals (65 days) but due to the small sample size (*n* = 35), it is not possible to draw definitive conclusions. This study adds to the scarce, but growing, evidence base on internationally comparable routes to diagnosis and time intervals and provides a strong basis for further investigation of the relationship between intervals and outcomes.

In all jurisdictions, we observed a similar and fairly typical symptom profile whether we analysed data from all patients or limited the analysis to the smaller cohort where we had data from both patients and their PCP. Differences in symptom reporting between patients and physicians were similar to that noted in previous reports by ICBP M4 for lung and colorectal cancers, with patients reporting fatigue as a key symptom more often than their PCP [20, 21]. In addition, difficulty eating and feeling full quickly was reported by considerably more OC patients (17%) compared to PCPs (1%). PCP access to specific guidance and/or pathways when managing non-specific but potentially serious symptoms (e.g. fatigue) varies across ICBP jurisdictions. Denmark most notably in response to earlier lower cancer survival rates, introduced in 2012 a pathway to manage patients presenting with non-specific symptoms and signs of cancer (serious non-specific symptoms and signs of cancer - cancer patient pathway; NSSC-CPP) [22]. It is possible that the adoption of NSSC-CPP was responsible for Denmark’s particularly short (median 1 day) primary-care interval. In addition to the Danish NSSC-CPP, efforts have been made in other ICBP jurisdictions (England, Scotland, Wales) to cater for this cohort of patients, although the impact upon interval length has not yet been quantified [23]. The management of patients with non-specific symptoms within primary care, requires greater investigation internationally.

### Comparisons with other studies

Patient reporting of symptoms is consistent with that previously reported for OC—primarily abdominal pain and distention, urogenital and gastrointestinal problems and fatigue [15, 24]. Our study also found a high proportion of patients (50%) reported ‘other’ symptoms which could not be reclassified into existing categories, further exploration of these symptoms is warranted.

The Australian Ovarian Cancer Study (2002–2005) found that 10% of women reported an incidental diagnosis, as did 13.5% of patients in a Manitoban study (2004–10) [25–27]. This is comparable to the 10–11% of patients across jurisdictions who were diagnosed due to ‘investigation for another problem’ in our study, although Northern Ireland and Norway differed somewhat to this estimate (2% and 18%, respectively). Care should be taken when interpreting this, however, due to the low number of respondents, particularly in Norway.

In our patient cohort, 9% of OC patients from England, and 13% internationally were diagnosed via emergency presentation. This compares with 26% of English OC patients in 2013 in a population-based study [28]. An Australian study showed 11.7% of OC patients had seen a hospital or emergency doctor before their diagnosis, however, only 4% had presented directly to a hospital or emergency department [27]. In our study, higher proportions (22–36%) of patients in Manitoba, Ontario and Northern Ireland presented to emergency departments. Our Northern Ireland data (24%) were similar to emergency admission (28%) reported in a 2010 Northern Ireland audit of OC patients [29]. Variation in methods, definitions of emergency diagnosis and sample sizes between studies are likely to have contributed to the differences between studies. The ICBP is currently exploring rates of emergency presentations between ICBP jurisdictions, which will add to our understanding of patient presentation and referral routes internationally.

There is limited comparable literature exploring time intervals. This is mainly due to variations in definitions and reporting of intervals. Interval lengths in a population-based, case-control study in Australian OC patients (2002–2005), reflect our findings. The interval defined by ‘first symptom to first medical practitioner consultation’ was under one month for 55.4% of their population—our median patient interval was close to or under one month for all jurisdictions [26]. Previous literature has shown that most patients receive a diagnosis within 90 days of presentation—in our study, in six of the nine participating jurisdictions, 75% of patients had diagnostic intervals of less than, or close to, 90 days (range 47–96) [25] [30]. Only Denmark, Northern Ireland and Manitoba had intervals of between 115 and 132 days.

Across all percentiles, the biggest variation between jurisdictions was seen in the diagnostic interval, with the median ranging from 25 days in Victoria to 68 days in Northern Ireland. Previous work has suggested that diagnostic delays in primary care, where 70% of our cohort presented, may be due to sub-optimal access to investigations rather than the physician recognising the need to investigate [31]. In our study, we observe substantial differences in the symptoms reported by patients and PCPs, which has also been previously shown for OC patients [5, 15].

Variation in treatment intervals may be due to variations in the way registry practices in the different countries determine the date of diagnosis. In Victoria, pathological confirmation (typically from a surgical procedure) is required to record the date at diagnosis, which can result in the date of diagnosis and date of treatment being the same [20]. This is likely to play a role in the treatment intervals for Victoria (and possibly Denmark, Northern Ireland and Manitoba) being 0 days. Exploration into this variation in practice is warranted to fully understand the impact upon international variation in treatment intervals. Other explanations include variation in the use of primary and interval debulking surgery across ICBP jurisdictions [32]. Differences have been reported in the rates of primary surgery (highest in Norway), as well as clinician-reported barriers to accessing optimal treatment. Danish clinicians most often reported having no barriers to accessing this care, which is in keeping with Denmark having comparably shorter treatment intervals in our study [32].

### Strengths and weaknesses

A key strength of this study is that it is the first to use an internationally standardised survey methodology to explore and compare key intervals from symptom onset to treatment start. The surveys drew on existing instruments and underwent cognitive testing, piloting, translation and adaption to ensure they were suitable for use in all participating countries and languages [11]. The use of data from cancer registries and other sources, alongside hierarchical data rules, allowed us to create as complete a record as possible of patient pathways to diagnosis and treatment. There are likely differences in questionnaire interpretation, patient characteristics and additional data availability, although our methodology and data analysis has sought to account for this where possible. We minimised recall bias [33] through the triangulation of different data sources and by ensuring that the patients received the questionnaire with a limited time window (3–6 months) after the cancer diagnosis [33]. The study was conducted in 2015 so it is worth considering that changes to service delivery across the jurisdictions may have happened in the interim that could affect the length of time intervals and routes to diagnosis reported here.

Most jurisdictions, except England and Denmark, were not able to recruit a sufficient number of patients to power this study. Response rates of eligible patients varied internationally, from 15.0% in Norway to 70.1% in Denmark. We were however not able to measure the direction of the resulting selection bias that differed across jurisdictions. The population over 80-year olds were particularly underrepresented among respondents. Also, of the identified eligible population, 42% had died within 6 months of diagnosis compared to 8% of the respondents. This significant difference suggests that it is likely that the differences in intervals that we have noted underestimate the magnitude of delays to diagnosis.

Participating women were comparable in several variables, such as self-assessed health state, comorbidity, and smoking, and are therefore unlikely to bias our results. As we were surveying in multiple languages across nine jurisdictions, we made a pragmatic decision to use a simple question to assess comorbidity rather than adopt a more systematic approach such as the Charlson comorbidity index. Differences in the classification systems for education and ethnicity may have introduced bias if included in the regression model and so were excluded. However, these are broadly comparable in the study population (primarily White, majority of low education). We note that there was a lack of diversity, a gap that needs to be addressed in future research. Our cohort is inevitably not representative of all OC patients, as women were only contacted 3–9 months post-diagnosis. Women who were diagnosed via emergency presentation had aggressive tumour morphology, advanced age and stage are underrepresented as these factors are associated with higher mortality in the first year after diagnosis [12, 34]. In addition, ‘healthy patient bias’ is likely to have contributed to a higher proportion of patients diagnosed with the early-stage disease in our study. For similar reasons, those reporting surgery (85% total, range 74–98%) were higher than observed in population-based studies of OC including the most recent from ICBP [2, 25]. We did not collect information on previous cancer or family history and were therefore unable to include information on genetic predisposition to OC.

## Conclusion

To the best of our knowledge, this is the first international study to compare routes to diagnosis and time intervals in recently diagnosed OC patients in a standardised way. It highlights key intervals in the diagnostic pathway where improvements could be made and provides the opportunity to consider the systems and approaches across different jurisdictions that could be associated with a more timely cancer diagnosis and treatment. A deeper exploration of the factors driving this variation and their potential impact on cancer outcomes is required. It would be important in any such future research to ensure that the ethnic diversity of the populations surveyed is reflected in the respondents.

## Supplementary information

Supplementary Materials

Reproducibility Checklist

## Data Availability

The datasets used and/or analysed during this study are available from the corresponding author on reasonable request.

## Code availability

Computer code used to generate statistical analyses is available from the authors upon request. Please contact the ICBP Programme management team, based at Cancer Research UK, with any queries (icbp@cancer.org.uk).
