# Supplementary material for: Diagnostic routes and time intervals for ovarian cancer in nine international jurisdictions; findings from the International Cancer Benchmarking Partnership (ICBP)
Source: Br J Cancer. 2022 May 26;127(5):844–54. doi: 10.1038/s41416-022-01844-0 (PMC9427750; doi:10.1038/s41416-022-01844-0)
Supplement: Supplementary file 1 — Supplementary Materials [file 41416_2022_1844_MOESM1_ESM.docx]

**Diagnostic routes and time intervals for ovarian cancer in nine international jurisdictions; findings from the International Cancer Benchmarking Partnership (ICBP) - Supplementary materials**

**Appendix A: Rules for missing, incomplete, multiple response and out of range data and for calculating intervals**

| 1. Oversampling/Participation in local screening trials 2. Exclude patients participated in local screen trials. |
| --- |
| 1. Language/Participation in study/Presence of cancer   Exclude patients who checked “No, I don’t understand the language” or “I don’t want to participate in this study” or “I don’t have cancer”. |
| 1. Survey responders 2. Exclude Patient/PCP/Specialist survey from the analysis, if it was not written by   Patient/PCP/Specialist (example: a medical oncologist completed a PCP survey);   1. In the case of duplicates, include only the first survey (example: 2 specialists completed surveys for the same patient). |
| 1. Age 2. Exclude patients with unknown age; 3. Exclude patients younger 40 years; 4. Use registry data, if Age is reported by both patient and registry. |
| 1. No cancer or Previous cancer in the same organ 2. Exclude patients with no cancer based on registry data; 3. Exclude patients with previous cancer in the same organ based on data from registry or free-text for Presentation in the patient survey. |
| 1. Date of consent   Exclude patients with date of consent which is unknown, before 01.01.2013 or in the future. |
| 1. Multiple responses to Dates   If multiple responses were given to the dates (of first symptom; screening; first presentation to primary care; referral; diagnosis; treatment start), then use the earliest date. |
| 1. Order of Dates   The dates must be in the following order –   1. First symptom; first presentation to Primary Care; referral; diagnosis; treatment start. 2. Screening; diagnosis; treatment start.   If not, check for mistakes. |
| 1. Date of first symptom   Date of first symptom is defined as date of first symptom from patient data. |
| 1. Date of first presentation   Date of first presentation to Primary Care is defined as (in the order of declining priority):   1. date of first presentation to Primary Care from PCP data; 2. date of first presentation to Primary Care and A&E from PCP data; 3. date of first presentation to Primary Care from patient data. |
| 1. Date of referral   Date of referral is defined as date of referral from PCP data. |
| 1. Date of diagnosis   *Definition*   1. If Registry reports both date of histological confirmation and date of confirming investigation, then use date of histological confirmation. 2. Date of diagnosis (based on patient data, PCP data, specialist data, registry data) is defined as (in the order of declining priority):  - date of diagnosis from registry; - date of histological confirmation (from specialist data, PCP data); - date of biopsy (from specialist data, PCP data); - date of confirming investigation (from specialist data, PCP data); - date of first hospital admission (from specialist data, PCP data); - date of MDT confirmation (from specialist data, PCP data); - date patient was told (from specialist data, PCP data); - other date of diagnosis (from specialist data, PCP data, patient data);   Choose a Date from a lower level of hierarchy, if the Date from a higher level is after the Date of  consent or more than 9 months (=271 days) before the Date of consent.  *Exclusion criteria*   1. Unknown date of diagnosis; 2. Date of diagnosis is after the date of consent; 3. Date of diagnosis is more than 9 months before the Date of consent. |
| 1. Date of treatment start 2. Date of treatment start from patient data is defined as the earliest of the treatment dates for Surgery, Chemo, Radio and Other; 3. Date of treatment start (based on registry data, specialist data, patient data) is defined as (in the order of declining priority):  - date of treatment start from registry data, - date of treatment start from specialist data, - date of treatment start from patient data, - anticipated date of treatment from patient data. |
| 1. Imputation of missing day in the date   Imputation rules for missing day (given month and year are known):   1. Set missing day to ‘16’; 2. Consider adjacent dates in a backwards order (from “Treatment” to “First symptom”). For each pair of such adjacent dates: If dates are not in a logical order (e.g. “Treatment” is before “Diagnosis”), but month and year are the same in both dates, and the day was imputed to ‘16’ in one of the dates:   - Recode the day imputed earlier to ‘16’ to the day from the adjacent date. |
| 1. Considering time   If patient gave multiple answers to the “How long did you have symptoms before contacting a doctor?” question, then use the option with the shortest time interval. |
| 1. Delay arranging appointment   If patient gave multiple answers to the “How long did it take to get an appointment with PCP?” question, then use the option with the shortest time interval. |
| 1. Duration of symptoms   If PCP gave multiple answers to the “Duration of symptoms” question, then use the option with the shortest time interval. |
| 1. Definition of Presentation 2. *Define Presentation within a Data Source (Patient, PCP)* 3. Review the free-text for Presentation (Patient, PCP) and re-code, if possible. 4. If PCP reports ‘VisitPCP and AE’ or ‘VisitPCP’ as Presentation and no symptoms, then check   Patient’s records. If Patient reports ‘Screening’ and no symptoms, then re-code Presentation  for this case as ‘Screening’.   1. If PCP reports ‘Screening’ as Presentation and at least one symptom (or “Duration of   Symptoms”, rule 19), then re-code Presentation to ‘Other non-screen-detected’-option.   1. If PCP reports ‘Other’ as Presentation and at least one symptom (or “Duration of   Symptoms”, rule 19), then re-code Presentation to ‘Other non-screen-detected’-option.   1. If Patient reports ‘Screening’ as Presentation and at least one symptom (or date of first   symptom), then re-code Presentation to ‘Other non-screen-detected’- option.   1. If Patient reports ‘Other’ as Presentation and at least one symptom (or date of first symptom   or “Considering time” (rule 17) or “Delay arranging appointment” (rule 18), then re-code  Presentation to ‘Other non-screen-detected’-option.   1. In the case of multiple Presentation responses (Patient, PCP sources) - use a single option (in   the order of declining priority):   1. ‘VisitPCP and AE’, 2. ‘VisitPCP´, ‘AE´ (if both ‘VisitPCP´ and ‘AE´ are given, then re-code as ‘VisitPCP and AE’), 3. ‘Other non-screen-detected’, 4. ‘Screening’, 5. ‘Investigation for another problem’ , 6. ’Other” 7. *Define Presentation from Alternative Data*   If Presentation hasn’t been reported in either of data sources, then define it as (in the order of declining priority):   1. ‘Other non-screen-detected ‘, if PCP reports at least one symptom (or “Duration of symptoms”, rule 19); 2. ‘Other non-screen-detected ‘, if Patient reports at least one symptom (or date of first symptom); 3. ‘Other non-screen-detected ‘, if Patient reports “Considering time” (rule 17) or “Delay arranging appointment” (rule 18) and no screening date; 4. ‘Screening’, if Patient reports screening date and no symptoms and no date of first symptom; 5. ‘Other non-screen-detected ‘, if cancer= CRC, jurisdiction=England, Age <58 or >76 years. 6. *Define Presentation from Data Source Hierarchy* 7. In all jurisdictions, except Sweden – use Presentation data from (in the order of declining   priority):  a) PCP data;  b) Patient data;   1. In Sweden – use Presentation data from Patient data. |
| 1. Patient interval   The Patient interval for non-screen-detected patients is defined as (in the order of declining priority):   1. “Date of first presentation to Primary Care” (rule 11) minus “Date of first symptom” (rule 10); 2. If the interval in (a) is unknown or negative: Calculate the interval as the low boundary of “Considering time” (rule 17) plus the low boundary of “Delay arranging appointment” (rule 18); 3. If the interval in (a) is unknown or negative and the interval in (b) is unknown: Calculate the interval as the low boundary of “Duration of symptoms interval” (rule 19). |
| 1. Primary Care interval   The Primary Care interval for non-screen-detected is defined as “Date of referral” (rule 12) minus “Date of first presentation to Primary Care” (rule 11). |
| 1. Diagnostic interval 2. The Diagnostic interval for non-screen-detected is defined as “Date of diagnosis” (rule 14) minus “Date of first presentation to Primary Care” (rule 11); 3. The Diagnostic interval for screen-detected patients is defined as “Date of diagnosis” (rule 14) minus “Date of screening” (rule 13). |
| 1. Treatment interval   The Treatment interval is defined as “Date of treatment start” (rule 15) minus “Date of diagnosis” (rule 14). |
| 1. Total interval 2. The Total interval for non-screen-detected patients is defined as “Date of treatment start” (rule 15) minus “Date of first symptom” (rule 10); 3. The Total interval for screen-detected patients is defined as “Date of treatment start” (rule 15) minus “Date of screening” (rule 13). |
| 1. Range of Time intervals   The time intervals (Patient, Primary Care, Diagnosis, Treatment, Total) must be in range 0-1 year.  If > 1 year: set the interval to 365 days  If negative: set the interval to 0.  For each jurisdiction calculate the number of imputations due to:   1. unknown day in a date (given known month and year); 2. very large(>1 year) interval; 3. negative interval. |
| 1. Number of visits   If patient gave multiple answers to the “Number of visits” questions, then use the option with a fewer number of visits. |
| 1. Specialist waiting time interval   If patient gave multiple answers to the ““How long did it take to get an appointment with specialist?” question, then use the option with the shortest time interval. |
| 1. Type of treatment   If patient ticked both “Yes” and “No” as answers to the “Type of treatment (Surgery, Chemotherapy, Radiotherapy)” questions, then choose “Yes” answer. |
| 1. Health state   If patient gave multiple answers to the “Health state” question, then use the option with a better health condition. |
| 1. Comorbidity 2. If patient ticked both “Yes” and “No” as answers to the “Comorbidity (Heart disease, Stroke, Lung disease, Diabetes)” questions, then choose “Yes” answer; 3. If both patient and PCP report “Comorbidity”, then use the PCP Data. |
| 1. Ethnicity 2. If patient didn’t report “Ethnicity”, then use the information from (in the order of declining priority):  - “Ethnicity_Other_Details”; - “Other main language spoken at home”; - “The main language spoken at home” (only for Victoria); - “The main language spoken at home is the chief one for this jurisdiction”=”Yes” given   “Main language spoken at home is other than the main one for this jurisdiction”=”No”;   1. Consider Ethnicity as unknown, if answers to the “Ethnicity” question are multiple and belong to   different categories ( ‘white’, ‘Asian’, ‘black’, ‘other’). |
| 1. Education   If patient gave multiple answers to the “Education” question, then use the option with a higher level of education. |
| 1. Smoking Current 2. If patient ticked both “Yes” and “No” as answers to the “Smoking Current” question, then use “Yes” answer; 3. If patient hasn’t ticked neither “Yes” nor “No, then consider this case as Unknown. |
| 1. Smoking Number   If patient reports “SmokingNumber” as text, then re-code using following rules:   1. Where there is a number smoked /day – accept number;   b) Where a range has been given – take the upper value;  c) Where patient has put 10+ or 20+ - capture this as 11 or 21;  d) Where number of cigarettes smoked in the past and currently being smoked are provided - average the numbers;  e) Non entries code as “.” ;  f) Non-smokers (eg, “nil”, “N/A“) are coded as “0”. |
| 1. Smoked ever 2. If patient ticked both “Yes” and “No” as answers to the “Smoking ever” question, then use “Yes” answer; 3. If patient hasn’t ticked neither “Yes” nor “No”: consider it as “Yes”, if patient is a current smoker (“Smoking_Current=”Yes””) or has specified a number of cigarettes (“SmokingNumber”>0). Otherwise consider this case as Unknown. 4. If patient has ticked “No”: recode it to “Yes”, if patient is a current smoker (“Smoking_Current=”Yes”). |
| 1. Nature of referral 2. Review free-text for “ Nature of referral” (PCP Data) and re-code, if possible; 3. In the case of multiple responses, use a single option as (in the order of declining priority):  - “Referral for immediate admission”; - “Urgent referral”; - “Less urgent referral”; - “General referral” ; - “No referral”; - “Other”. |
| 1. Refer Public or Private 2. If PCP ticked both “Public” and “Private” as answers to the “Refer Public or Private” question, then use “Private” answer; 3. If PCP hasn’t ticked neither “Public” nor “Private”, then consider this case as Unknown. |
| 1. Type of referral   If specialist gave multiple responses to the “How was the patient referred…” question, then use a single option (in the order of declining priority):   - “Screening”; - “Respiratory clinic”; - “General surgery clinic”; - “General gynaecology”;   - “Specialist/consultant”;  - “PCP”;  - “Other”. |
| 1. First Attendance Place   If specialist gave multiple responses to the “First Attendance Place” question, then consider this case as Unknown. |
| 1. Stage-TNM 2. If specialist gave multiple responses to the “Stage_TNM” question, then use the highest category; 3. If registry gave multiple responses to the “Stage_TNM”, then use a single option (in the order of declining priority):  - stage at time of diagnosis - stage at surgery - stage at oncology  1. If “Stage_TNM” is reported by both the specialist and registry, then use the registry data; 2. If “Stage_TNM” is unknown or “not able to stage”, then use “Stage_Duke” or “Stage_Figo”. |
| 1. Stage_Dukes 2. If specialist gave multiple responses to the “Stage_Dukes’” question, then use the highest category; 3. If “Stage_Dukes” is reported by both the specialist and registry, then use the registry data. |
| 1. Stage_Figo 2. If specialist gave multiple responses to the “Stage_Figo” question, then use the highest category; 3. If “Stage_Figo” is reported by both the specialist and registry, then use the registry data. |

**Appendix B: Ovarian Cancer Questionnaires**

1. Patient

# International Cancer Benchmarking Partnership Module 4

# Patient questionnaire

# Ovarian Cancer

*Thank you very much for taking the time to fill in this questionnaire – it should take about 20 minutes to complete. We are sending the questionnaire to a large sample of people who we understand have had a diagnosis of ovarian cancer. If this has been sent to you in error and you do not have cancer, please do not continue and return the documents in the prepaid envelope.*

*Our aim is to gain a better understanding of the process by which people have their cancer diagnosed. We would also like to find out more about the symptoms they experience (if any), and the pathway they follow from start of symptoms to treatment of their cancer. This will help in identifying ways in which cancers can be diagnosed quickly and effectively. Thank you once again for your time.*

**This information is confidential and will not be passed to anyone involved in your treatment**

**Name:**

**Date of Birth:**

**Address:**

**Consent form**

Please read the consent form and sign your name and date at the bottom.

If you require any clarification, please do not hesitate to ring the study team members [x] on [local phone number]. Their contact details can also be found on the information sheet.

**Please be reassured that your responses are completely confidential and will not be passed to anyone involved in your treatment. For the purposes of the study it is important that you agree to consent to all the statements listed below.**

- I confirm that I have read the attached information sheet and I understand why the research is being done
- I am willing for the team to request information from my GP and hospital doctors which is relevant to the audit as described in the information sheet
- I give permission for my details (name, address) to be given to the cancer registry (NHS Information Centre for Health and Social Care) for follow up.
- I agree for the information I have provided and any other relevant information from my medical records to be stored as described in the information sheet under the custodianship of University College London
- I consent to sharing of coded data which contains no personal identifiers between researchers, some of whom are located outside the European Union.
- I consent for use of my data if I become mentally incapacitated during the course of the project

**I agree to all the statements listed above and consent to participate in the study.**

Name (Please print) _______________________

Signature:____________________________ Date: _______________________

If we have any questions, may we phone you for clarification? (Please **✓**) **Yes No**

If **Yes**, please provide your telephone number: _______________________

Is this survey in a language you can understand? (Please **✓**)

| - Yes | → | Please continue |
| --- | --- | --- |
| - No, I do not understand the language | → | Please do not continue and simply return the questionnaire in the free post envelope |
| - No, I do not understand the language,   but I had help from a friend or family member to fill in this questionnaire | → | Please continue |
| Other reasons you may have for not completing the study: (Please **✓**) | | |
| - I do not want to participate in this study | → | Please do not continue and simply return the questionnaire in the free post envelope |
| - I do not have cancer | → | Please do not continue and simply return the questionnaire in the free post envelope |

1. Please can you confirm the details of your GP/GP practice (name, practice address – as best as you can remember): We appreciate that you may have more than one GP involved in your care – in which case, we are interested in the GP you would say provides the majority of your care, particularly relating to the cancer you’ve had diagnosed.

Name of doctor……………………………………………………………………………..

Name of practice……………………………………………………………………………

Address…………………………………………………………………………………………..

Postcode………………………………………………………………………………………...

Town....................................................................................................

1. Which of the following best describes the events which led to your diagnosis of cancer? (**please ✓only one answer**)

- I had symptoms/I noticed a bodily change and went to see a doctor (e.g. GP).
- I had symptoms/I noticed a bodily change and went/was taken to Accident and Emergency (A&E)
- I had seen a doctor/GP with symptoms, but went/was taken to Accident and Emergency (A&E) when things worsened
- I was being investigated by my doctor(s) for another problem during which time the cancer was discovered.
- Other (please describe**)**

.............................................................................................................................

.............................................................................................................................

.............................................................................................................................

1. The following health concerns or symptoms are commonly experienced with ovarian cancer.

- Pelvic or abdominal pain
- Abdominal distension / increased abdominal size / persistent bloating
- Difficulty eating and feeling full quickly (early satiety)
- Increased urinary urgency and/or frequency
- Bleeding after the menopause
- Change in bowel habit
- Loss of appetite
- Unexplained weight loss
- Fatigue/tiredness

Please write down **all** health concerns or symptoms you may have had before contacting a doctor. It does not matter if they are not included in the list above:

| ***Please write your health concern(s)/ symptom(s) in the boxes below:*** |
| --- |
| *1)* |
| *2)* |
| *3)* |
| *4)* |
| *5)* |
| *6)* |

- This is not applicable to me (eg I did not have any symptoms)

1. Please write down your best estimate of the date you noticed the first of these health concern(s)/symptom(s). *If you cannot remember the exact date, you can fill in the month and the year.*

| *Day (optional) month year*  -  D  D  M  M  Y  -  Y  Y  Y |
| --- |

- This is not applicable to me (eg I had no symptoms)

1. Approximately how long did you have **health concern(s) or symptom(s)** before contacting a doctor? (Please think of the first visit to the doctor, not re-visits after that). **Please ✓ only one answer.**

- Less than 1 week
- 1-2 weeks
- 3-4 weeks
- 5-7 weeks
- 2-5 months
- 6-12 months
- More than 12 months
- This is not applicable to me (eg I had no symptoms)

6a. Once you **contacted** a practice/office about your health concern(s) or symptom(s), how long did it take to get an appointment with a doctor? (Please think of the first visit to the doctor, to discuss your health concern or symptom). **Please ✓ only one answer.**

- Same day/next day
- Within 1 week
- 1-2 weeks
- 3-4 weeks
- Longer

If there was no waiting time (eg you went/were taken to A&E), please tick this box

- This is not applicable to me (eg I had no symptoms)

6b. What was the date you first saw your doctor about your health concern(s) or symptom(s)? *If you cannot remember the exact date, you can fill in the month and the year.*

*Day (optional) month year*

-

D

D

M

M

Y

-

Y

Y

Y

- This is not applicable to me (eg I had no symptoms)

1. How many times did you visit the following for the investigation of your symptoms before your cancer was diagnosed?

|  | Please write down the number of visits |
| --- | --- |
| GP |  |
| Hospital |  |
| Consultant/specialist outside of a hospital |  |
| - This is not applicable to me (eg I had no symptoms) | |

8a. After your doctor referred you to a specialist, how long did it take you to get an appointment? **Please ✓ only one answer.**

- Less than 1 week
- 1-2 weeks
- 3-4 weeks
- 5-7 weeks
- 2-5 months
- 6-12 months
- More than 12 months
- This is not applicable to me (eg my doctor did not refer me)

8b. What was the date of your first appointment with a doctor, involved in investigating and/or treating your cancer, to whom you were referred? *If you cannot remember the exact date, you can fill in the month and the year.*

*Day (optional) month year*

-

DDDD

D

M

M

Y

-

Y

Y

Y

D

- This is not applicable to me (eg my doctor did not refer me)

1. What was the date you were told you had cancer? *If you cannot remember the exact date, you can fill in the month and the year.*

*Day (optional) month year*

-

DDDD

D

M

M

Y

-

Y

Y

Y

D

1. Have you had any of the following treatments for your cancer yet? If so, please can you estimate the date this treatment started? **Please ✓ all that apply.**

*If you cannot remember the exact date, you can fill in the month and the year.*

|  | **Type of treatment** | | **Date of treatment**  **(give first date if you had more than one)** |
| --- | --- | --- | --- |
| a. | Surgery | - Yes - No | *Day (optional) month year*  -  D  D  M  M  Y  -  Y  Y  Y |
| b. | Chemotherapy | - Yes - No | *Day (optional) month year*  -  D  D  M  M  Y  -  Y  Y  Y |
| c. | Radiotherapy | - Yes - No | *Day (optional) month year*  -  D  D  M  M  Y  -  Y  Y  Y |
| d. | Other |  | *Day (optional) month year*  -  D  D  M  M  Y  -  Y  Y  Y |
|  | Please specify: | |  |
| e. | Treatment not started yet |  | *Day (optional) month year*  -  D  D  M  M  Y  -  Y  Y  Y |

1. Who is the consultant doctor who has taken responsibility for diagnosing and or/treating your cancer?

Name of consultant:

Hospital name:

Hospital department:

Please can you answer some more general questions about your health? It will help us in interpreting your responses to this questionnaire to know about your general health and other health problems you may have had in the past.

1. Looking back to the 2 years before you were diagnosed with cancer, would you say your general health was:

**Please ✓ only one answer.**

- Very good
- Good
- Fair
- Poor
- Very poor

1. Have you been treated before for any of the conditions below?

**Please ✓ tick ‘yes’ or ‘no’ for each condition:**

Heart disease….Yes No

Stroke……………..Yes No

Lung disease……Yes No

Diabetes………….Yes No

**Finally, a little more information about you.** The information you provide below will help us to analyse the results of the survey in more detail.

1. Which of these best describes your ethnic group? (please tick as appropriate. If you are descended from more than one ethnic or racial group, please tick the group you consider you belong to or tick 'Any other ethnic group?

| - White | - Chinese | - Black - Caribbean | - Black - African |
| --- | --- | --- | --- |
| - Black - other | - Indian | - Pakistani | - Bangladeshi |
| - Any other ethnic group__________________________________________________ | | | |

1. What is the main language spoken in your home?

- English
- Other, please specify…………………………

1. What is the highest level of education you have achieved?

**Please ✓ only one answer.**

- Finished school at or before the age of fifteen
- Completed GCSEs, O-levels or equivalent
- Completed A Levels or equivalent
- Completed further education but not a degree
- Completed a Bachelor’s degree / Masters degree / PhD
- Other, please specify.…………………………

1. Have you ever smoked cigarettes, including hand-rolled ones, pipes or cigars?

- Yes
- No

1. Are you a current smoker, smoking either cigarettes, including hand-rolled ones, pipes or cigars?

- Yes
- No

1. If you are a current smoker or have smoked in the past, how many cigarettes, including hand-rolled ones, pipes or cigars on average do you smoke/have you smoked per day?

Number per day:____________________________________________________

**Further comments**

**Please add anything else that you would like to tell us about your cancer diagnosis or treatment.**

**Thank you very much for taking the time to complete this questionnaire.**

1. GP

# ICBP Module 4

# Ovarian cancer primary care audit

Thank you very much for agreeing to fill in this questionnaire. As part of an international study examining differences in cancer survival, we are sending the questionnaire to health care providers of a sample of consented patients with cancer.

Our aim is to gain a better understanding of the process by which people have their cancer diagnosed – the symptoms they experience, and the pathway they follow from onset of symptoms to treatment of their cancer. This will help in identifying ways in which cancers can be diagnosed and treated quickly and effectively. Thank you once again for your time.

| **Please can you refer to your patient’s notes in completing the questionnaire as this will help in obtaining accurate data on time points.** |
| --- |

**ID-number: Jurisdiction-ID + Patient-ID:__________________________________**

If you would prefer to return this questionnaire without the patient details, please tear off along the dotted line

...............................................................................................................................

**Patient information**

**ID-number: Jurisdiction-ID + Patient-ID:__________________________________**

Full name:__________________________________________________________________

Address, including postcode:____________________________________________________

___________________________________________________________________________

___________________________________________________________________________

Postcode:__________________

-

D

M

M

Y

-

Y

Y

Y

D

Date of birth:

**1. Duration of symptoms**

Please estimate how long your patient had symptom(s), attributable to ovarian cancer, before attending your practice (or other health service).

We appreciate that identifying a ‘date of first symptom’ is not always straightforward – particularly when there are multiple and/or chronic symptoms. Nevertheless, we hope you can provide a ‘best estimate’.

| ***Estimate of symptom duration (please tick one):*** | ***What were the symptoms?***  *Please describe:* |
| --- | --- |
| - Less than 1 week |  |
| - 1 to 4 weeks |  |
| - 5 to 7 weeks |  |
| - 2-5 months |  |
| - 6-12 months |  |
| - More than 12 months |  |
| - Not possible to estimate |  |
| - No symptoms (e.g. screen detected cancers) |  |

**2. Pathway of presentation**

**2.1 Through what route did the patient first present? Please tick one:**

| - Your patient first presented to primary care (either in-hours or out-of-hours) | Please can you provide your best approximation of the date of this **primary care** visit  -  D  D  M  M  Y  -  Y  Y  Y |
| --- | --- |
| - Your patient presented straight to A&E (with or without your involvement) |  |
| - Your patient first presented to primary care, but then at a later date presented to A&E as an emergency (with or without your involvement) | Please can you provide your best approximation of the date of the **primary care** visit  -  D  D  M  M  Y  -  Y  Y  Y |
| - Other – please describe |  |

**3. Date you ordered any investigations in response to symptom(s).**

We are interested in any kind of test (e.g. imaging etc) that you may have ordered. **Please tick all that apply.**

- Specific blood test for ovarian cancer (CA-125)
- Pelvic ultrasound
- Other (please specify)

**4. Date of referral to specialist medical services**

At what date did you ***first*** refer the patient to hospital or another specialist *transferring the responsibility for on-going investigation/treatment* to other medical services?

-

D

D

M

M

Y

-

Y

Y

Y

**5. Nature of this referral**

**5.1 Do you know the date that the patient was seen for this referral?**

-

D

D

M

M

Y

-

Y

Y

Y

- Yes, please provide the date:
- No

**5.2 If you did make a referral to specialist services, which of the following best describes the nature/characteristics of this referral?**

| - Emergency admission: a referral to A&E (or equivalent) for immediate admission |
| --- |
| - An urgent referral for assessment of cancer symptoms/signs/test results (Note this will be within 2 weeks for England/Wales) |
| - A less urgent referral in which cancer is raised as a possibility (Note this will be greater than 2 weeks for England/Wales) |
| - A more general referral for investigation and assessment without cancer mentioned |
| - No referral was made |
| - Other – please describe |

**5.3 Would you say this patient’s diagnostic pathway was conducted predominantly in the public or private system?**

- Public healthcare system
- Private healthcare system

**6. Date of ovarian cancer diagnosis**

This can be decided in different ways. Please provide whichever of the following dates you have to hand:

| - Date of histological confirmation [ideal] | -  D  D  M  Y  Y  Y  Y  M |
| --- | --- |
| - Date results of investigation confirming cancer received | -  D  D  M  Y  Y  Y  Y  M |
| - Date patient was told | -  D  D  M  M  Y  -  Y  Y  Y |
| - Date of biopsy | -  D  D  M  M  Y  -  Y  Y  Y |
| - Date patient was first admitted to hospital because of the malignancy | -  D  D  M  M  Y  -  Y  Y  Y |
| - Other (please specify) | -  D  D  M  M  Y  -  Y  Y  Y |

**7. Additional information**

Finally, we are interested to know what other conditions your patient has, and the severity/impact of these conditions

Have you and/or any of your partners treated this patient (or has the patient been to hospital) for any of the following conditions? **Please tick all that apply:**

Cardiovascular disease…..Yes No

Stroke………………………Yes No

Lung disease………………Yes No

Diabetes……………………Yes No

| **Are there any other comments you would like to make about this patient?** |
| --- |

Name (and title):____________________________

Signature:_________________________________

Date:_____________________________________

**Thank you for taking the time to complete this questionnaire**

1. Cancer treatment specialist (CTS)

# ICBP Module 4

# Specialist care audit

# Ovarian Cancer

| Thank you very much for agreeing to fill in this questionnaire – it should take about 10 minutes to complete. As part of an international study examining differences in cancer survival, we are sending the questionnaire to health care providers of a sample of patients with cancer.  Your patient……………………………………………………………………….  is participating in the study.  Our aim is to gain a better understanding of the process by which people have their cancer diagnosed – the symptoms they experience, and the pathway they follow from onset of symptoms to treatment of their cancer. We hope you can help us with information on this patient’s cancer journey once they were referred to specialist cancer services. This will help in identifying ways in which cancers can be diagnosed and treated quickly and effectively.  **Thank you once again for your time** |
| --- |

If you would prefer to return this questionnaire without the patient details, please tear off along the dotted line

| **Please can you refer to your patient’s it’s notes in completing the questionnaire, as this will help in obtaining accurate data on time points.** |
| --- |

**Patient information**

**ID-number: Jurisdiction-ID + Patient-ID:__________________________________**

Full name:__________________________________________________________________

Address:____________________________________________________

___________________________________________________________________________

___________________________________________________________________________

Postcode:__________________

-

D

M

M

Y

-

Y

Y

Y

D

Date of birth:

**1. Date patient first attended hospital/specialist services related to their cancer diagnosis**

We appreciate this date can at times be difficult to identify, particularly when there have been multiple visits in the lead up to a definitive diagnosis. Put another way, it’s the date that the hospital/specialist service *assumed responsibility for on-going investigation/treatment* for your patient

-

D

M

M

Y

-

Y

Y

Y

D

**2. How was the patient referred to the hospital/specialist services related to their cancer diagnosis?**

**Was it through a:**

- GP referral
- Medical specialist/Consultant referral
- Referral from general gynaecology
- Other referral

**3. Where did this first contact/appointment happen?**

Which of the following best describes where this first contact/appointment took place?

- Emergency department (’A&E’)
- Gynaecology oncology outpatient
- Gynaecology general outpatient department
- Oncology general outpatient department
- Medical outpatient department, please specify which department
- Surgical outpatient department, please specify which department
- Other - please specify

**4. Date of diagnosis**

This can be decided in different ways. Please complete as many of the following dates as possible.

- Date of histological confirmation (ideal)

-

D

M

M

Y

-

Y

Y

Y

D

-

D

M

M

Y

-

Y

Y

Y

D

- Date results of investigation confirming

cancer received

-

D

M

M

Y

-

Y

Y

Y

D

- Date patient was told
- Date of biopsy

-

D

M

M

Y

-

Y

Y

Y

D

- Date patient was first admitted to

-

M

M

Y

-

Y

Y

Y

D

D

hospital because of the malignancy

- Date of MDT confirmation of diagnosis

M

M

Y

-

Y

Y

Y

D

D

-

- Other (please specify)

-

D

M

M

Y

-

Y

Y

Y

D

.................................................................

**5. Date treatment for the cancer commenced**

Based on your records, when would you say that any treatment specifically targeting the patient’s cancer started?

-

D

M

M

Y

-

Y

Y

Y

D

**6. Additional information**

Please can you provide any further information on the patient’s cancer:

| **TNM, please tick as appropriate:**   - 0 - I - IIA - IIB - IIC - IIIA - IIIB - IIIC - IV - **Not able to stage** | **­­­­­­­­­­­­­­­­­­­­­­­FIGO, please tick as appropriate:**   - 1A - 1B - 1C - 2A - 2B - 2C - 3A - 3B - 3C - 4 |
| --- | --- |

**6.1 Histological subtype:**

- Serous
- Mucinous
- Endometrioid
- Clear cell
- Borderline Serous
- Borderline Mucinous
- Primary non-epithelial malignant tumours (please specify)
- Other (please specify)

| **Any further comments:** |
| --- |

Name (and title):____________________________

Signature: _________________________________

Date:_____________________________________

Are you a (please tick below):

- Surgeon
- Medical Oncologist
- Clinical Oncologist
- Clinical Nurse Specialist
- Other, please specify.......................................................

**Thank you for taking the time to fill in this questionnaire**

**Table 1:** Patient flow from identification to analyses for all jurisdictions and overall

| **Jurisdiction** | **Denmark** | | **England** | | **Victoria** | | **Scotland** | | **Ontario** | | **Wales** | | **N Ireland** | | **Manitoba** | | **Norway** | | | **Total** | | |
| --- | --- | --- | --- | --- | --- | --- | --- | --- | --- | --- | --- | --- | --- | --- | --- | --- | --- | --- | --- | --- | --- | --- |
| Start date | 28-10-2013 | | 01-11-2013 | | 01-07-2013 | | 01-12-2013 | | 30-04-2014 | | 04-10-2013 | | 06-08-2013 | | 01-05-2013 | | 01-09-2014 | | |  | |  |
|  | n | (%) | n | (%) | n | (%) | n | (%) | n | (%) | n | (%) | n | (%) | n | (%) | | n | (%) | n | (%) | |
| **Eligible patients^a, b^** | **388** | **(100%)** | **988** | **(100%)** | **303** | **(100%)** | **442** | **(100%)** | **437** | **(100%)** | **439** | **(100%)** | **143** | **(100%)** | **143** | **(100%)** | | **360** | **(100%)** | **3,204** | **(100%)** | |
|  |  |  |  |  |  |  |  |  |  |  |  |  |  |  |  |  | |  |  |  |  | |
| **Packs sent to PCP^c, d^** |  |  | **988** | **(100%)** |  |  | **442** | **(100%)** |  |  | **439** | **(100%)** |  |  |  |  | |  |  | **1,869** | **(100%)** | |
| *Pack not forwarded by PCP* |  |  | *89* | *(9.0%)* |  |  | *53* | *(12.0%)* |  |  | *80* | *(18.2%)* |  |  |  |  | |  |  | *222* | *(11.9%)* | |
| *Unsure if pack forwarded by PCP* |  |  | *289* | *(29.3%)* |  |  | *81* | *(18.3%)* |  |  | *128* | *(29.2%)* |  |  |  |  | |  |  | *498* | *(26.6%)* | |
| **Patients contacted by PCP^c, d^** |  |  | ***610*** | ***(61.7%)*** |  |  | ***308*** | ***(69.7%)*** |  |  | ***231*** | ***(52.6%)*** |  |  |  |  | |  |  | **1,149** | **(61.5%)** | |
|  |  |  |  |  |  |  |  |  |  |  |  |  |  |  |  |  | |  |  |  |  | |
| **Patients approached directly^c^** | **388** | **(100%)** |  |  | **237** | **(78.2%)** |  |  | **385** | **(88.1%)** |  |  | **141** | **(98.6%)** | **105** | **(73.4%)** | | **311** | **(86.4%)** | **1,567** | **(48.9%)** | |
| *Patient died* |  |  |  |  |  |  |  |  | *n≤5* |  |  |  | *n≤5* |  |  |  | |  |  | *7* | *(0.4%)* | |
| *Unknown address* |  |  |  |  |  |  |  |  | *22* | *(3.5%)* |  |  |  |  |  |  | |  |  | *22* | *(1.4%)* | |
| *Other* |  |  |  |  |  |  |  |  | *24* | *(3.8%)* |  |  |  |  |  |  | |  |  | *24* | *(1.5%)* | |
|  |  |  |  |  |  |  |  |  |  |  |  |  |  |  |  |  | |  |  |  |  | |
| **Patient responses**  **(% of eligible patients)** | **271** | **(69.8%)***^e^* | **256** | **(25.9%)** | **136** | **(44.9%)** | **140** | **(31.7%)** | **109** | **(24.9%)** | **98** | **(22.3%)** | **95** | **(66.4%)** | **56** | **(39.2%)***^e^* | | **51** | **(14.2%)***^e^* | **1,213** | **(37.8%)***^e^* | |
|  |  |  |  |  |  |  |  |  |  |  |  |  |  |  |  |  | |  |  |  |  | |
| **Patient responses**  **(% of contacted)^f^** | **271** | **(69.8%)***^e^* | **256** | **(42.0%)** | **136** | **(57.4%)** | **140** | **(45.5%)** | **109** | **(32.6%)** | **98** | **(42.4%)** | **95** | **(68.3%)** | **56** | **(53.3%)***^e^* | | **51** | **(16.4%)***^e^* | **1,213** | **(44.7%)***^e^* | |
| *Excluded^g^* | *n≤5* |  | *26* | *(10.2%)* | *9* | *(6.6%)* | *39* | *(27.9%)* | *10* | *(9.2%)* | *8* | *(8.2%)* | *10* | *(10.5%)* | *n≤5* |  | | *n≤5* |  | *102* | *(8.5%)* | |
|  |  |  |  |  |  |  |  |  |  |  |  |  |  |  |  |  | |  |  |  |  | |
| **Included in analyses** |  |  |  |  |  |  |  |  |  |  |  |  |  |  |  |  | |  |  |  |  | |
| **Patient surveys**  (% of respondent patients) | **271** | **(100%)** | **230** | **(89.8%)** | **127** | **(93.4%)** | **101** | **(72.1%)** | **99** | **(90.8%)** | **90** | **(91.8%)** | **85** | **(89.5%)** | **56** | **(100%)** | | **51** | **(100%)** | **1,110** | **(91.5%)** | |
|  |  |  |  |  |  |  |  |  |  |  |  |  |  |  |  |  | |  |  |  |  | |
| **PCP surveys**  (% of patient surveys) | **198** | **(73.1%)** | **179** | **(77.8%)** | **76** | **(59.8%)** | **76** | **(75.2%)** | **38** | **(38.4%)** | **63** | **(70.0%)** | **77** | **(90.6%)** | **38** | **(67.9%)** | | **10** | **(19.6%)** | **755** | **(68.0%)** | |
|  |  |  |  |  |  |  |  |  |  |  |  |  |  |  |  |  | |  |  |  |  | |
| **Specialist surveys**  (% of patient surveys) | **29**^h^ | **(10.7%)** | **141** | **(61.3%)** | **55** | **(43.3%)** | **58** | **(57.4%)** | **20** | **(20.2%)** | **58** | **(64.4%)** | **n/a**^h^ | | **n/a**^i^ | **9** | | | **(17.6%)** | **370** | **(38.2%)**^j^ | |

^a^ Eligible according to protocol: i.e. woman. 40 years or more. consented to participate, and diagnosed with ovarian cancer incl. cancer in the fallopian tube (ICD-10 code: C57.0-C57.9 or C56.9; behaviour code ICD-O 3) but not synchronous primary ovarian cancer and with no prior history of ovarian cancer who were alive at time of identification.

^b^ In some jurisdictions some ‘eligible’ patients had pre-opted out from being contacted and a small number where PCP information was not available.

^c^ Percentages of eligible patients.

^d^ Known number of contacted patients. i.e. packs known to be forwarded by PCP to patients.

^e^ Excluding patients with previous ovarian cancer, unknown date of consent, ineligibility or sampled for local purposes in Denmark, Manitoba and Norway (n=9).

^f^Percentages of patients contacted by PCP (see note d) for Wales. England and Scotland or percentages of patients contacted directly by a registry excl. non-accessible patients (all other jurisdictions).

^g^ Exclusion reasons: previous ovarian cancer, unknown date of consent, ineligibility, or sampled for local purposes

^h^ Data obtained from registries instead: N Ireland from the N Ireland Cancer Registry, supported by extracts from clinical datasets; Denmark from the Danish Gynaecological Cancer Database

^i^ Data not collected in this jurisdiction. ^j^ Denominator = total number of analysed cases excl. patients from Manitoba & N Ireland. Some numbers are not shown due to the data protection regulations.

Table 2: Respondent patients in relation to identified patients for each ICBPM4 jurisdiction

|  | **Wales** | | | | | | **England** | | | | | **Scotland** | | | | | **N Ireland** | | | | | **Denmark** | | | | |
| --- | --- | --- | --- | --- | --- | --- | --- | --- | --- | --- | --- | --- | --- | --- | --- | --- | --- | --- | --- | --- | --- | --- | --- | --- | --- | --- |
|  | Identified | | | Respondents | | p-value | Identified | | Respondents | | p-value | Identified | | Respondents | | p-value | Identified | | Respondents | | p-value | Identified | | Respondents | | p-value |
| No of women | 439 | | | 98 | |  | 808 | | 205 | |  | 360 | | 112 | |  | 349 | | 85 | |  | 489 | | 272 | |  |
| Age Group |  | | | | |  |  |  |  |  |  |  |  |  |  |  |  |  |  |  |  |  |  |  |  |  |
| < 40 | 38 | | (8.7%) | 8 | (8.2%) | 0.589 | 49 | (6.1%) | n≤5 |  | **0.006** | 0 | (0%) | 0 | (0%) | 0.237 | 0 | (0%) | 0 | (0%) | **0.017** | 0 | (0%) | 0 | (0%) | 0.492 |
| 40-49 | 52 | | (11.8%) | 10 | (10.2%) |  | 78 | (9.7%) | 17 | (8.3%) |  | 46 | (12.8%) | 15 | (13.4%) |  | 49 | (14%) | 8 | (9.4%) |  | 47 | (10%) | 28 | (10.3%) |  |
| 50-59 | 85 | | (19.4%) | 21 | (21.4%) |  | 166 | (20.5%) | 61 | (29.8%) |  | 88 | (24.4%) | 30 | (26.8%) |  | 86 | (24.6%) | 29 | (34.1%) |  | 75 | (16%) | 53 | (19.5%) |  |
| 60-69 | 110 | | (25.1%) | 29 | (29.6%) |  | 257 | (31.8%) | 65 | (31.7%) |  | 106 | (29.4%) | 39 | (34.8%) |  | 93 | (26.6%) | 29 | (34.1%) |  | 155 | (33.1%) | 88 | (32.4%) |  |
| 70-79 | 89 | | (20.3%) | 22 | (22.4%) |  | 183 | (22.6%) | 47 | (22.9%) |  | 95 | (26.4%) | 24 | (21.4%) |  | 68 | (19.5%) | 17 | (20%) |  | 133 | (28.4%) | 81 | (29.8%) |  |
| 80-89 | 48 | | (10.9%) | 8 | (8.2%) |  | 64 | (7.9%) | 13 | (6.3%) |  | 23 | (6.4%) | n≤5 |  |  | 45 | (12.9%) | n≤5 |  |  | 51 | (10.9%) | 20 | (7.4%) |  |
| 90+ | 8 | | (1.8%) | 0 | (0%) |  | 11 | (1.4%) | 0 | (0%) |  | n≤5 |  | n≤5 |  |  | 8 | (2.3%) | 0 | (0%) |  | 7 | (1.5%) | n≤5 |  |  |
| Missing | 9 | | (2.1%) | 0 | (0%) |  | 0 | (0%) | n≤5 |  |  | n≤5 |  | 0 | (0%) |  | 0 | (0%) | n≤5 |  |  | 0 | (0%) | n≤5 |  |  |
| **Dead at** |  | |  |  |  |  |  |  |  |  |  |  |  |  |  |  |  |  |  |  |  |  |  |  |  |  |
| 3 months | 40 | | (17.5%) | 0 | (0%) | 0.310 | 13 | (11.4%) | n≤5 |  | 0.216 | 9 | (7.1%) | 0 | (0%) | 0.087 | n/a |  | n/a |  |  | 45 | (19%) | n≤5 |  | **<0.001** |
| 6 months | 54 | | (23.7%) | 0 | (0%) |  | 31 | (27.2%) | n≤5 |  |  | 25 | (19.8%) | n≤5 |  |  | n/a |  | n/a |  |  | 54 | (22.8%) | n≤5 |  |  |
| 9 months | 62 | | (27.2%) | n≤5 |  |  | 34 | (29.8%) | 7 | (36.8%) |  | 39 | (31%) | n≤5 |  |  | n/a |  | n/a |  |  | 65 | (27.4%) | 10 | (27%) |  |
| 12 months | 72 | | (31.6%) | n≤5 |  |  | 36 | (31.6%) | 9 | (47.4%) |  | 53 | (42.1%) | 11 | (68.8%) |  | n/a |  | n/a |  |  | 73 | (30.8%) | 24 | (64.9%) |  |
| **Tumour stage – TNM & FIGO** |  | |  |  |  |  |  |  |  |  |  |  |  |  |  |  |  |  |  |  |  |  |  |  |  |  |
| I | 56 | | (12.8%) | 15 | (15.3%) | 0.050 | 232 | (28.7%) | 51 | (24.9%) | **0.035** | 96 | (28.7%) | 32 | (30.5%) | 0.709 | 111 | (31.8%) | 24 | (28.2%) | **0.036** | 41 | (18.9%) | 74 | (27.2%) | **<0.001** |
| II | 12 | | (2.7%) | n≤5 |  | 0.426* | 62 | (7.7%) | 23 | (11.2%) | **0.045*** | 36 | (10.7%) | 15 | (14.3%) | 0.709* | 24 | (6.9%) | n≤5 |  | 0.177* | 11 | (5.1%) | 10 | (3.7%) | 0.472* |
| III | 52 | | (11.8%) | 22 | (22.4%) |  | 296 | (36.6%) | 92 | (44.9%) |  | 137 | (40.9%) | 40 | (38.1%) |  | 139 | (39.8%) | 48 | (56.5%) |  | 58 | (26.7%) | 99 | (36.4%) |  |
| IV | 36 | | (8.2%) | n≤10 |  |  | 136 | (16.8%) | 25 | (12.2%) |  | 66 | (19.7%) | 18 | (17.1%) |  | 34 | (9.7%) | 6 | (7.1%) |  | 31 | (14.3%) | 44 | (16.2%) |  |
| Missing | 283 | | (64.5%) | 50 | (51%) |  | 82 | (10.1%) | 14 | (6.8%) |  | 0 | (0%) | 0 | (0%) |  | 41 | (11.7%) | n≤5 |  |  | 76 | (35%) | 45 | (16.5%) |  |
|  |  | |  |  |  |  |  |  |  |  |  |  |  |  |  |  |  |  |  |  |  |  |  |  |  |  |
|  | **Norway** | | | | | | **Manitoba** | | | | | **Ontario** | | | | | **Victoria** | | | | | **Total** | | | | |
|  | Identified | | | Respondents | | p-value | Identified | | Respondents | | p-value | Identified | | Respondents | | p-value | Identified | | Respondents | | p-value | Identified | | Respondents | | p-value |
| No of women | 360 | | | 54 | |  | 175 | | 57 | |  | 437 | | 110 | |  | 408 | | 116 | |  | 3825 | | 1109 | |  |
| **Age group** | |  |  |  |  |  |  |  |  |  |  |  |  |  |  |  |  |  |  |  |  |  |  |  |  |  |
| < 40 | | 0 | (0%) | 0 | (0%) | 0.274 | 0 | (0%) | 0 | (0%) | 0.064 | 0 | (0%) | 0 | (0%) | 0.630 | 0 | (0%) | 0 | (0%) | **0.019** | 87 | (2.3%) | 10 | (0.9%) | **<0.001** |
| 40-49 | | 37 | (10.3%) | n≤5 |  |  | 29 | (16.6%) | 8 | (14%) |  | 68 | (15.6%) | 20 | (18.2%) |  | 49 | (12%) | 16 | (13.8%) |  | 455 | (12%) | 127 | (11.5%) |  |
| 50-59 | | 85 | (23.6%) | 16 | (29.6%) |  | 49 | (28%) | 20 | (35.1%) |  | 122 | (27.9%) | 35 | (31.8%) |  | 103 | (25.2%) | 37 | (31.9%) |  | 859 | (22.6%) | 302 | (27.2%) |  |
| 60-69 | | 115 | (31.9%) | 18 | (33.3%) |  | 47 | (26.9%) | 17 | (29.8%) |  | 129 | (29.5%) | 34 | (30.9%) |  | 113 | (27.7%) | 41 | (35.3%) |  | 1125 | (29.6%) | 360 | (32.5%) |  |
| 70-79 | | 74 | (20.6%) | 12 | (22.2%) |  | 25 | (14.3%) | 12 | (21.1%) |  | 84 | (19.2%) | 16 | (14.5%) |  | 100 | (24.5%) | 18 | (15.5%) |  | 851 | (22.4%) | 249 | (22.5%) |  |
| 80-89 | | 43 | (11.9%) | n≤5 |  |  | 21 | (12%) | 0 | (0%) |  | 32 | (7.3%) | n≤5 |  |  | 43 | (10.5%) | n≤5 |  |  | 370 | (9.7%) | 55 | (5%) |  |
| 90+ | | 6 | (1.7%) | n≤5 |  |  | n≤5 |  | 0 | (0%) |  | n≤5 |  | 0 | (0%) |  | 0 | (0%) | 0 | (0%) |  | 48 | (1.3%) | 6 | (0.5%) |  |
| Missing | | 0 | (0%) | 0 | (0%) |  | n≤5 |  | 0 | (0%) |  | n≤5 |  | n≤5 |  |  | 0 | (0%) | n≤5 |  |  | 9 | (0.2%) | 0 | (0%) |  |
| **Dead at** | |  |  |  |  |  |  |  |  |  |  |  |  |  |  |  |  |  |  |  |  |  |  |  |  |  |
| 3 months | | 20 | (50%) | 0 | (0%) | 0.173 | 21 | (52.5%) |  | (0%) | 0.124 | 14 | (19.2%) | 0 | (0%) | 0.270 | 15 | (30%) | 0 | (0%) | 0.081 | 177 | (19.5%) | 0 | (0%) | **<0.001** |
| 6 months | | 6 | (15%) | 0 | (0%) |  | 11 | (27.5%) | n≤5 |  |  | n≤15 |  | n≤5 |  |  | 8 | (16%) | 0 | (0%) |  | 203 | (22.4%) | 8 | (8.4%) |  |
| 9 months | | 8 | (20%) | n≤5 |  |  | n≤5 |  | 0 | (0%) |  | n≤5 |  | n≤5 |  |  | 14 | (28%) | n≤5 |  |  | 228 | (25.1%) | 28 | (29.5%) |  |
| 12 months | | 6 | (15%) | n≤5 |  |  | n≤5 |  | n≤5 |  |  | 41 | (56.2%) | 9 | (81.8%) |  | 13 | (26%) | n≤5 |  |  | 300 | (33%) | 59 | (62.1%) |  |
| **Tumour stage – TNM & FIGO** | |  |  |  |  |  |  |  |  |  |  |  |  |  |  |  |  |  |  |  |  |  |  |  |  |  |
| I | | n/a |  | n/a |  |  | 32 | (22.9%) | 13 | (26%) | 0.750 | 51 | (11.7%) | 16 | (14.7%) | 0.920 | n/a |  | n/a |  |  | 523 | (21.9%) | 193 | (23.6%) | **<0.001** |
| II | | n/a |  | n/a |  |  | 24 | (17.1%) | 7 | (14%) | 0.750* | 24 | (5.5%) | n≤5 |  | 0.834* | n/a |  | n/a |  |  | 157 | (6.6%) | 51 | (6.2%) | 0.104* |
| III | | n/a |  | n/a |  |  | 60 | (42.9%) | 24 | (48%) |  | 135 | (30.9%) | 34 | (31.2%) |  | n/a |  | n/a |  |  | 740 | (31%) | 319 | (38.9%) |  |
| IV | | n/a |  | n/a |  |  | 24 | (17.1%) | 6 | (12%) |  | 41 | (9.4%) | n≤10 |  |  | n/a |  | n/a |  |  | 302 | (12.6%) | 99 | (12.1%) |  |
| Missing | | n/a |  | n/a |  |  | 0 | (0%) | 0 | (0%) |  | 186 | (42.6%) | 45 | (41.3%) |  | n/a |  | n/a |  |  | 668 | (27.9%) | 157 | (19.2%) |  |

n/a=data not available

P-values are based on the Pearson’s chi-squared test.

* excluding Missing data

Some numbers are not shown due to the data protection regulations.

**Table 3.** Histological subtype reported by Cancer Treatment Specialists for ovarian cancer patients n (%)^a^.

|  | **Denmark**^b^ | **England** | **Victoria** | **Scotland** | **Ontario** | **Wales** | | **N Ireland**^c^ | | **Norway** | | **Total** |
| --- | --- | --- | --- | --- | --- | --- | --- | --- | --- | --- | --- | --- |
| No of women | 271 | 141 | 55 | 58 | 20 | 58 | | 85 | | 9 | | 697 |
| **Histological subtype** |  |  |  |  |  |  | |  | |  | |  |
| Serous | 19 (7.0) | 67 (47.5) | 44 (80) | 33 (56.9) | 15 (75.0) | 33 (56.9) | | 62 (72.9) | | 6 (66.7) | | 279 (40.0) |
| Mucinous/  Endometrioid/  Clear cell | n≤5 | 41 (29.1) | 6 (10.9) | 19 (32.8) | n≤5 | 10 (17.2) | | 20 (23.5) | | n≤5 | | 100 (14.4) |
| Borderline | n≤5 | n≤5 | n≤5 | n≤5 | n≤5 | 6 (10.3) | | n≤5 | | n≤5 | | 12 (1.7) |
| Non-epithelial | n≤5 | n≤5 | n≤5 | n≤5 | n≤5 | n≤5 | | n≤5 | | n≤5 | | 6 (0.9) |
| Other | n≤5 | 13 (9.2) | n≤5 | n≤5 | n≤5 | 8 (13.8) | | n≤5 | | n≤5 | | 29 (4.2) |
| Missing | 249 (91.9) | 13 (9.2) | n≤5 | n≤5 | n≤5 | n≤5 n≤5 | | | n≤5 | |  | 271 (38.9) |
|  | | |  |  |  | |  | |  | |  |  |

^a^This information was only available on the subgroup of patients where their treating specialist completed a questionnaire or information was provided by from the registry. Manitoba is not included as there was no information available

^b^. Data obtained from the Danish Gynaecological Cancer Database

^c^. Data obtained from the N Ireland Cancer Registry, supported by extracts form clinical databases.

Some numbers are not shown due to the data protection regulations.

**Table 4:** Patient reported interval for arranging an appointment with a PCP for ovarian cancer patients (figures are n (%)).

|  | **Denmark** | **England** | **Victoria** | **Scotland** | **Ontario** | **Wales** | **N Ireland** | **Manitoba** | **Norway** | **Total** |
| --- | --- | --- | --- | --- | --- | --- | --- | --- | --- | --- |
|  | (N=271) | (N=230) | (N=127) | (N=101) | (N=99) | (N=90) | (N=85) | (N=56) | (N=51) | (N=1110) |
| no waiting time e.g. went to A&E | 16 (6) | 14 (6) | 10 (8) | n≤5 | 10 (10) | 8 (9) | 6 (7) | n≤5 | n≤5 | 73 (7) |
| 0-6 days | 193 (71) | 171 (74) | 92 (72) | 77 (76) | 58 (58) | 55 (61) | 58 (68) | 29 (52) | 18 (35) | 751 (68) |
| 1-4 weeks | 33 (12) | 34 (15) | 13 (10) | 10 (10) | 17 (17) | 19 (21) | 14 (16) | 9 (16) | 14 (27) | 163 (15) |
| more than 4 weeks | n≤5 | n≤5 | n≤5 | n≤5 | n≤5 | n≤5 | n≤5 | n≤5 | n≤5 | 20 (2) |
| not applicable to me | 15 (6) | n≤5 | n≤5 | n≤5 | 6 (6) | n≤5 | 0 | n≤5 | 14 (27) | 52 (5) |
| missing | n≤15 | n≤5 | n≤5 | 7 (7) | n≤5 | n≤5 | n≤5 | 11 (20) | n≤5 | 51 (5) |

Some numbers are not shown due to the data protection regulations.

**Table 5a.** First symptoms experienced by patients and presenting symptoms noted by PCP for eligible patients. All figures are n (%) unless otherwise states.

|  | **Denmark** | **England** | **Victoria** | **Scotland** | **Ontario** | **Wales** | **N Ireland** | **Manitoba** | **Norway** | **Total** |
| --- | --- | --- | --- | --- | --- | --- | --- | --- | --- | --- |
| **Symptoms experienced by all patient** | (N=271) | (N=230) | (N=127) | (N=101) | (N=99) | (N=90) | (N=85) | (N=56) | (N=51) | (N=1110) |
| swelling in abdomen, increased abdomen size/ bloating/unexplained weight gain | 112 (41) | 132 (57) | 62 (49) | 51 (51) | 66 (67) | 44 (49) | 53 (62) | 35 (63) | 21 (41) | 576 (52) |
| unexplained pain (abdomen, stomach or pelvis) | 95 (35) | 92 (40) | 58 (46) | 40 (40) | 48 (48) | 39 (43) | 42 (49) | 23 (41) | 13 (25) | 450 (41) |
| fatigue | 57 (21) | 86 (37) | 36 (28) | 33 (33) | 34 (34) | 24 (27) | 30 (35) | 19 (34) | 6 (12) | 325 (29) |
| changes in urinary pattern | 54 (20) | 87 (38) | 30 (24) | 26 (26) | 28 (28) | 25 (28) | 25 (29) | 19 (34) | 10 (20) | 304 (27) |
| changes in bowel habits | 47 (17) | 57 (25) | 36 (28) | 25 (25) | 33 (33) | 19 (21) | 18 (21) | n≤15 | n≤5 | 251 (23) |
| difficulty eating, and feeling full quick | n≤10 | 58 (25) | 24 (19) | 22 (22) | 28 (28) | 15 (17) | 13 (15) | 14 (25) | n≤5 | 188 (17) |
| Other | 107 (39) | 131 (57) | 62 (49) | 53 (52) | 47 (47) | 52 (58) | 50 (59) | 34 (61) | 23 (45) | 559 (50) |
| no symptoms | 33 (12) | 7 (3) | 8 (6) | 12 (12) | 6 (6) | 7 (8) | n≤5 | n≤5 | 9 (18) | 91 (8) |
| Missing | 14 (5) | n≤5 | n≤5 | n≤5 | 0 | 0 | n≤5 | n≤5 | n≤5 | 29 (3) |
| **Number of symptoms per patient** |  |  |  |  |  |  |  |  |  |  |
| Median (IQI) | 2 (1,3) | 3 (2,5) | 2 (1,4) | 3 (1,4) | 3 (1,4) | 3 (1,4) | 3 (2,5) | 3 (2,5) | 2 (1,3) | 2 (1,4) |
| **Symptoms reported by PCP** | (N=166) | (N=144) | (N=72) | (N=68) | (N=34) | (N=49) | (N=0) | (N=33) | (N=7) | (N=573) |
| swelling in abdomen (increased abdomen size) / bloating/unexplained weight gain | 26 (16) | 40 (28) | 14 (19) | 16 (24) | 6 (18) | 17 (35) | n/a | 9 (27) | 0 | 128 (22) |
| unexplained pain (abdomen, stomach or pelvis) | 39 (23) | 50 (35) | 39 (54) | 22 (32) | n≤15 | 18 (37) | n/a | 15 (45) | n≤5 | 199 (35) |
| fatigue | n≤5 | n≤5 | n≤5 | n≤5 | 0 | 0 | n/a | 0 | 0 | 17 (3) |
| changes in urinary pattern | 11 (7) | 12 (8) | 7 (10) | n≤5 | n≤5 | n≤5 | n/a | n≤5 | 0 | 44 (8) |
| changes in bowel habits | 15 (9) | 21 (15) | 12 (17) | 13 (19) | n≤5 | n≤5 | n/a | n≤5 | 0 | 71 (12) |
| difficulty eating, and feeling full quick | n≤5 | n≤5 | 0 | n≤5 | 0 | n≤5 | n/a | n≤5 | 0 | 7 (1) |
| other | 56 (34) | 63 (44) | 30 (42) | 33 (49) | 12 (35) | 18 (37) | n/a | n≤15 | n≤5 | 226 (39) |
| no symptoms | 0 | n≤5 | n≤5 | n≤5 | n≤5 | n≤5 | n/a | n≤5 | 0 | 14 (2) |
| missing | 59 (36) | 17 (12) | n≤5 | 12 (18) | n≤5 | n≤5 | n/a | n≤5 | n≤5 | 107 (19) |
| **Number of symptoms per patient** |  |  |  |  |  |  |  |  |  |  |
| Median (IQI) | 1 (1,2) | 1 (1,2) | 1 (1,2) | 2 (1,2) | 1 (1,2) | 1 (1,2) | n/a | 2 (1,2) | 1 (1,1) | 1 (1,2) |
| **Of symptoms reported by PCP, those considered suggestive of ovarian cancer (%)** | | | | | |  |  |  |  |  |
| Based on list of symptoms in original GSI | 36 | 59 | 69 | 57 | 53 | 63 | n/a | 55 | 29 | 53 |
| Based on list of symptoms in NG122 guidelines | 50 | 68 | 75 | 65 | 65 | 67 | n/a | 58 | 29 | 62 |

IQI=inter-quartile interval; GSI= Goff Symptom Index (GSI); NG122= NICE. Ovarian cancer: recognition and initial management NICE Guidelines. Clinical guideline 122

Some numbers are not shown due to the data protection regulations.

**Table 5b.** First symptoms experienced by patients and presenting symptoms noted by PCP for the 573 eligible patients with both data sources. All figures are n (%) unless otherwise stated

|  | **Denmark** | **England** | **Victoria** | **Scotland** | **Ontario** | **Wales** | **N Ireland** | **Manitoba** | **Norway** | **Total** |
| --- | --- | --- | --- | --- | --- | --- | --- | --- | --- | --- |
| **Symptoms experienced by patient** | (N=166) | (N=144) | (N=72) | (N=68) | (N=34) | (N=49) | (N=0) | (N=33) | (N=7) | (N=573) |
| swelling in abdomen, increased abdomen size/ bloating/unexplained weight gain | 74 (45) | 84 (58) | 34 (47) | 40 (59) | 23 (67) | 28 (57) | n/a | 17 (52) | n≤5 | 301 (53) |
| unexplained pain (abdomen, stomach or pelvis) | 63 (38) | 55 (38) | 36 (50) | 30 (44) | 19 (56) | 22 (45) | n/a | 15 (45) | n≤5 | 242 (42) |
| fatigue | 37 (22) | 52 (36) | 15 (21) | 24 (35) | 10 (29) | 10 (20) | n/a | 12 (36) | 0 | 160 (28) |
| changes in urinary pattern | 32 (19) | 56 (39) | 20 (28) | 18 (26) | 9 (26) | 17 (35) | n/a | 11 (33) | n≤5 | 164 (29) |
| changes in bowel habits | 31 (19) | 37 (26) | 21 (29) | 17 (25) | 14 (41) | 11 (22) | n/a | 6 (18) | n≤5 | 139 (26) |
| difficulty eating, and feeling full quick | n≤5 | 40 (28) | 15 (21) | 13 (19) | 9 (26) | 9 (18) | n/a | 9 (27) | 0 | 99 (17) |
| Other | 19 (11) | 43 (30) | 15 (21) | 15 (22) | n≤5 | 8 (16) | n/a | 10 (30) | n≤5 | 115 (20) |
| no symptoms | 13 (8) | n≤5 | n≤5 | 7 (10) | 0 | n≤5 | n/a | n≤5 | 0 | 33 (8) |
| missing | 8 (5) | 0 | n≤5 | 0 | 0 | 0 | n/a | n≤5 | n≤5 | 13 (2) |
| **Number of symptoms per patient** |  |  |  |  |  |  |  |  |  |  |
| Median (IQI) | 2 (1,3) | 3 (2,5) | 2 (1,4) | 3 (1,4) | 3 (2,5) | 3 (2,4) | n/a | 3 (2,5) | 2 (2,3) | 2 (1,4) |

Some numbers are not shown due to the data protection regulations.

*The ICBP Module 4 working Group (full list):*

- Alina Zalounina Falborg, Statistician, Research Unit for General Practice, Department of Public Health, Aarhus University, Bartholins Allé 2, 8000 Aarhus C, Denmark;
- Andriana Barisic, Research Associate, Department of Prevention and Cancer Control, Cancer Care Ontario, 620 University Avenue, Toronto, Ontario, M5G 2L7, Canada;
- Anna Gavin, Director, Northern Ireland Cancer Registry, Centre for Public Health, Queen's University Belfast, Mulhouse Building, Mulhouse Road, Belfast, BT12 6DP, United Kingdom;
- Anne Kari Knudsen, Administrative leader, Department of Cancer Research and Molecular Medicine, Norwegian University of Science and Technology, 7489 Trondheim, Norway;
- Breann Hawryluk, Project Planning Coordinator, Department of Patient Navigation, Cancer Care Manitoba, 675 McDermot Street, Winnipeg, Manitoba, Canada;
- Chantelle Anandan, Research Fellow, Centre for Population Health Sciences, University of Edinburgh, Doorway 1, Medical Quad Teviot Place, Edinburgh, EH8 9DX, United Kingdom;
- Conan Donnelly, Research Fellow, Centre for Public Health, Queen's University Belfast, Mulhouse Building, Mulhouse Road, Belfast, BT12 6DP, United Kingdom;
- David H Brewster, Scottish Cancer Registry, Information Services Division, NHS National Services Scotland, Gyle Square, 1 South Gyle Crescent, Edinburgh, EH12 9EB, United Kingdom;
- David Weller, James Mackenzie Professor of General Practice, Centre for Population Health Sciences, University of Edinburgh, Doorway 1, Medical Quad Teviot Place, Edinburgh, EH8 9DX, United Kingdom;
- Donna Turner, Epidemiologist/Provincial Director, Population Oncology, Cancer Care Manitoba, 675 McDermot Street, Winnipeg, Manitoba, Canada;
- Elizabeth Harland, Project Coordinator, Department of Epidemiology and Cancer Registry, CancerCare Manitoba, 675 McDermot Street, Winnipeg, Manitoba;
- Eva Grunfeld, Director, Knowledge Translation Research Network Health Services Research Program, Ontario Institute for Cancer Research; Professor and Vice Chair Research, Department of Family and Community Medicine, University of Toronto, 500 University Avenue, Toronto, Ontario, M5G 1V7, Canada;
- Evangelia Ourania Fourkala, Research Associate, Gynaecological Cancer Research Centre, Women's Cancer, Institute for Women's Health, University College London, United Kingdom;
- Henry Jensen, Research fellow, Research Unit for General Practice, Department of Public Health, Aarhus University, Bartholins Allé 2, 8000 Aarhus C, Denmark;
- Jackie Boylan, Research Fellow, Centre for Public Health, Queen's University Belfast, Mulhouse Building, Mulhouse Road, Belfast, BT12 6DP, United Kingdom;
- Jacqueline Kelly, Tumour Verification Officer, Northern Ireland Cancer Registry, Centre for Public Health, Queen’s University Belfast, Mulhouse Building, Mulhouse Road, Belfast, BT12 6DP, United Kingdom;
- Kerry Moore, Research Fellow, Centre for Public Health, Queen's University Belfast, Mulhouse Building, Mulhouse Road, Belfast, BT12 6DP, United Kingdom;
- Maria Rejmyr Davis, Head, Southern Sweden Regional Cancer Center, Medicon Village, Scheelevägen 8, building 404, 223 81 Lund, Sweden;
- Martin Malmberg, MD PhD, Senior Consultant, Department of Oncology, Lund University Hospital, SE-221 85 Lund, Sweden;
- Mats Lambe, Professor of Medical Epidemiology, Regional Cancer Center Uppsala and Department of Medical Epidemiology and Biostatics, Karolinska Institutet, SE-171 77 Stockholm, Sweden;
- Oliver Bucher, Epidemiologist, Department of Epidemiology and Cancer Registry, CancerCare Manitoba, 675 McDermot Street, Winnipeg, Manitoba;
- Peter Vedsted, Professor, Research Unit for General Practice, Department of Public Health, Aarhus University, Bartholins Allé 2, 8000 Aarhus C, Denmark;
- Rebecca Bergin, Senior Research Officer/ PhD Candidate, Centre for Behavioural Research in Cancer, 615 St Kilda Rd, Melbourne, Victoria, 3004, Australia;
- Rebecca-Jane Law, Research Project Support Officer, North Wales Centre for Primary Care Research, Bangor University, Gwenfro Units 4-8, Wrexham Technology Park, Wrexham, LL13 7YP, United Kingdom;
- Richard D Neal, Professor of Primary Care Oncology, Academic Unit of Primary Care, Leeds Institute of Health Sciences, University of Leeds, Leeds LS2 9NL, United Kingdom;
- Sigrun Saur Almberg, Researcher, Department of Cancer Research and Molecular Medicine, Faculty of Medicine, Norwegian University of Science and Technology (NTNU), N-7491 Trondheim, Norway;
- Therese Kearney, Research fellow, Northern Ireland Cancer Registry, Centre for Public Health, Queen’s University Belfast, Mulhouse Building, Mulhouse Road, Belfast, BT12 6DP, United Kingdom;
- Jatinderpal Kalsi, Project Manager, Gynaecological Cancer Research Centre, Women's Cancer, Institute for Women's Health, University College London, United Kingdom;
- Victoria Cairnduff, Statistician, Northern Ireland Cancer Registry, Centre for Public Health, Queen’s University Belfast, Mulhouse Building, Mulhouse Road, Belfast, BT12 6DP, United Kingdom;
- Victoria Hammersley, Researcher, Centre for Population Health Sciences, University of Edinburgh, Doorway 1, Medical Quad Teviot Place, Edinburgh, EH8 9DX, United Kingdom;
- Victoria White, Deputy Director, Centre for Behavioral Research in Cancer, Cancer Council Victoria, 615 St Kilda Road, Melbourne, Victoria, 3004, Australia;
- Usha Menon, Professor of Gynaecological Oncology and Head, Gynaecological Cancer Research Centre, Women's Cancer, Institute for Women's Health, University College London, United Kingdom;
- Yulan Lin, Postdoc, Department of Cancer Research and Molecular Medicine, Faculty of Medicine, Norwegian University of Science and Technology (NTNU), N-7491 Trondheim, Norway
